# Structural and Biochemical Characterization of *Fusobacterium nucleatum* Enoyl-ACP Reductase II (FabK) Reveals the Basis for Bacterial Species-Specific Inhibition

**DOI:** 10.1021/acsbiomedchemau.5c00199

**Published:** 2025-11-19

**Authors:** Kristiana Avad, Osama Alaidi, Destiny Okpomo, Fahad Bin Aziz Pavel, Darcy Doran, Madeline Matheson, Dianqing Sun, Julian Hurdle, Kirk E. Hevener

**Affiliations:** † Department of Pharmaceutical Sciences, College of Pharmacy, 12326 University of Tennessee Health Science Center, Memphis, Tennessee 38163, United States; ‡ Department of Translational Medical Sciences, Center for Inflammatory and Infectious Diseases, Institute of Biosciences and Technology, Texas A&M Health Science Center, Houston, Texas 77030, United States; § Department of Pharmaceutical Sciences, The Daniel K. Inouye College of Pharmacy, 14679 University of Hawaii at Hilo, Hilo, Hawaii 96720, United States

**Keywords:** fatty acid synthesis, enoyl ACP reductase, fabK, narrow-spectrum, antibacterial, Fusobacterium nucleatum, periodontal disease

## Abstract

*Fusobacterium nucleatum* is a Gram-negative anaerobic bacterium ubiquitous in the oral cavity and increasingly recognized for its involvement in diverse clinical conditions, including periodontal disease, inflammatory bowel disease, premature birth, and several forms of cancer. These associations highlight the need for narrow-spectrum antibacterial agents directed against *F. nucleatum* to avoid disruption of beneficial microflora and limit the rise of antibiotic resistance. Recent studies have identified the fusobacterial fatty acid synthesis pathway (FAS-II) enzyme, enoyl-acyl carrier protein (ACP) reductase, *Fn*FabK, as an essential and promising target for selective antibacterial intervention. However, there is a lack of detailed structural information, which has hindered the validation of *Fn*FabK’s druggability and the discovery of new inhibitors. Here, we present a comprehensive characterization of *Fn*FabK, including its cocrystal structure solved at 2.25 Å resolution and its biochemical and biophysical interactions with a series of potent small-molecule inhibitors. Our analyses revealed that these inhibitors display low to submicromolar activity against *Fn*FabK, with notable selectivity and differential activity when tested against FabK homologues from other bacterial pathogens. Importantly, the unique structural features of the *Fn*FabK active site, elucidated through these crystallographic studies, provide a mechanistic basis for species-specific inhibition. These findings not only validate *Fn*FabK as a druggable target but also furnish critical insights into the design of next-generation narrow-spectrum antibacterial agents.

## Graphical abstract

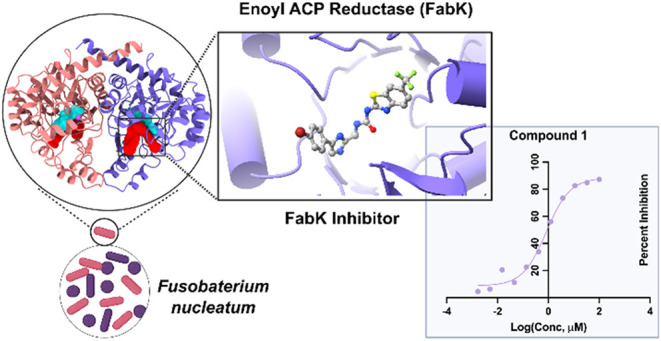

## Introduction

*Fusobacterium nucleatum* is a Gram-negative anaerobic bacillus that resides in the oral cavity.^1^ It acts as an opportunistic pathogen in multiple diseases, such as colorectal cancer, adverse pregnancy outcomes, and most notably periodontitis. In these disease settings, the pathogenesis of *F. nucleatum* benefits from dysbiosis and underlying inflammation. While antibiotics have been used in treating *F. nucleatum*-associated infections, these agents may further exacerbate dysbiosis. Thus, there is a need for antibiotics with narrow-spectrum activity to avoid dysbiosis to minimize collateral damage to the beneficial microbiota, while eradicating infecting pathogens.^2^ One pathway that has proven successful and is thus worth exploring is the fatty acid synthesis II (FAS-II) pathway.^3,4^ The species-specific distribution of FabK, along with its distinct structural and mechanistic properties compared to the FabI/L/V isozymes, makes it an appealing target for narrow-spectrum antibacterial development (Table S1).^5,6^ A key concern in targeting the FAS-II pathway is whether bacteria can bypass the inhibition of the enoyl-ACP reductase step by importing exogenous fatty acids from the host. This issue has been a subject of considerable debate.^7–9^ Certain Gram-positive bacteria, such as *Streptococcus* species, can circumvent the FAS-II pathway depending on their transcriptional regulation systems. Consequently, FAS-II inhibition is only effective against select Gram-positive organisms, such as *Staphylococcus aureus*.^10^

In contrast, Gram-negative bacteria like *F. nucleatum* have distinct fatty acid requirements—particularly for lipid A synthesis in the outer membrane—that cannot be fulfilled by host-derived fatty acids.^11,12^ The lipid A biosynthetic pathway depends on β-hydroxy fatty acids, whose hydroxyl groups are essential for acylation reactions. Notably, the acyltransferases involved in lipid A formation specifically utilize acyl carrier protein (ACP) thioester substrates produced by the FAS-II system.^13^ Therefore, supplementation with hydroxy fatty acids does not rescue growth, making inhibition of the FAS-II pathway a viable strategy for combating Gram-negative infections. With regard to *F. nucleatum*, we recently reported that this organism encodes the enoyl-ACP reductase (ENR) enzyme FabK that performs a critical, rate-limiting step of its fatty acid synthesis II (FAS-II) pathway.^14^ Genetic validation demonstrated that *Fn*FabK was essential and that FabK inhibitors diminished the growth of *F. nucleatum* while showing a narrow-spectrum activity.

FabI, FabL, and FabV belong to the short-chain dehydrogenase reductase (SDR) class that favors the use of NAD­(P)H in the reaction. This class, which is known to possess a characteristic Rossman fold structural motif,^15,16^ has successfully been targeted with several known enoyl-ACP reductase I (FabI) inhibitors (such as *isoniazid* and *triclosan*) that currently are on the market, and afabicin, which is near entering the market. Bioinformatics analysis, however, showed that *F. nucleatum* expresses FabK as the sole ENR isozyme.^14^ Using gene silencing, previous studies successfully showed that the expression of FabK is essential for *F. nucleatum*.^14^ These studies showed that in the absence of the *fabk* gene, even with supplementation of exogenous fatty acids, *F. nucleatum* growth was inhibited, thus demonstrating the essentiality of *Fn*FabK for the overall bacterial survival. These findings suggested FabK as an antibacterial target for the treatment of *F. nucleatum*.

Unlike the other ENRs, FabK possesses a TIM barrel protein structural motif and is a flavoenzyme that requires flavin mononucleotide (FMN) and nicotinamide adenine dinucleotide (phosphate) (NAD­(P)­H) for its enzymatic function.^17^ FabK also has a very distinct enzymatic mechanism, relative to the other Fab enzymes in this class. It employs a bi-bi double displacement (ping pong) catalytic mechanism as opposed to the ordered-sequential mechanism that is observed in the SDR class of enzymes.^18,19^ Furthermore, while FabK is commonly found in anaerobic pathogenic organisms, most commensal organisms do not solely rely on or express FabK (Table S1). This further supports the potential for the development of highly selective antibacterial agents. To date, we have been able to identify several FabK selective inhibitors, and we discovered that several of these inhibitors exhibit a varying degree of activity against the different FabK enzymes from bacterial species including *Clostridium difficile* (*Cd*FabK)^20–23^ and *F. nucleatum* (*Fn*FabK).^14^ Since the active sites of these enzymes are nearly identical with respect to residue conservation (*see* the Supporting Information), this posed the immediate question of what was the structural basis for the activity differences observed. Although several FabK structures have been solved, including the apo structure of *Porphyromonas gingivalis* FabK and an inhibitor-bound structure of *C. difficile* FabK,^5,24^ the answer to the question was not immediately apparent.

To determine the structural basis of the observed activity differences, we determined the cocrystal structure of the *Fn*FabK enzyme bound to a selective and potent inhibitor. Further, we characterized small-molecule inhibitors of *Fn*FabK using biochemical and biophysical methods, which highlighted key structural features needed for inhibition. We discuss herein how the overall morphology of the FabK activity site partly explains the varying activity of the FabK inhibitors against *Cd*FabK and *Fn*FabK. The same compounds displayed no observable activity against the FabI isozyme present in *S. aureus* (*Sa*FabI), indicating its high selectivity for FabK enzymes. These observations are expected to facilitate antibacterial discovery targeting the FabK enzyme and further support the rational design of more species-selective antibacterial agents that can reduce dysbiosis and overcome antibiotic resistance.

## Results

Our study had two objectives: First, to test the *Fn*FabK inhibitory activity of a set of benzothiazole derivatives that have been previously shown to inhibit *Cd*FabK.^22,23^ Second, to determine the structural basis for the species-specific difference in FabK activity observed for several of these compounds. Accordingly, we investigated the enzymatic activity of *Fn*FabK using an optimized fluorescence intensity (FI) assay in a dose-dependent manner to determine the apparent *K*_m_ of the substrates and the IC_50_ values of inhibitors. Compounds were then tested in our thermo-FMN (thermal shift) assay to confirm on-target binding.^22^ Lastly, the binding affinity was then determined for the best compound (**1**) via a dose response thermos-FMN assay. We then solved and analyzed the cocrystal structure of *Fn*FabK bound to the latter compound.

### Double Substrate Inhibition Observed with FnFabK

The optimal concentrations of both the NADH cofactor and butenoyl-CoA (also known as crotonyl-CoA) substrate for enzyme activity were determined by calculating their apparent *K*_m_ values (*K*_m_^app^). The *K*_m_^app^ of NADH was determined using 1.5-fold serial dilutions of NADH while holding butenoyl-CoA fixed at 375 μM (Figure 1A). In a similar fashion, butenoyl-CoA concentrations were varied using a 1.5-fold serial dilution while holding NADH constant at 160 μM (Figure 1B). When plotting the data, substrate inhibition patterns for both cofactor and substrate were noted, as previously observed in our previous studies with *C. difficile* FabK enzyme.^20^ Therefore, we used substrate inhibition nonlinear regression models to determine the *K*_m_^app^ values for each.^25^ A fit of the data to typical Michaelis–Menten kinetic models is shown in Figure 1 for comparison. Using substrate inhibition models, the *K*_m_^app^ values were determined to be 147 and 166 μM for NADH and butenoyl-CoA, with K_i_ values of 210 μM and 2.2 mM, respectively.

**Figure 1:**
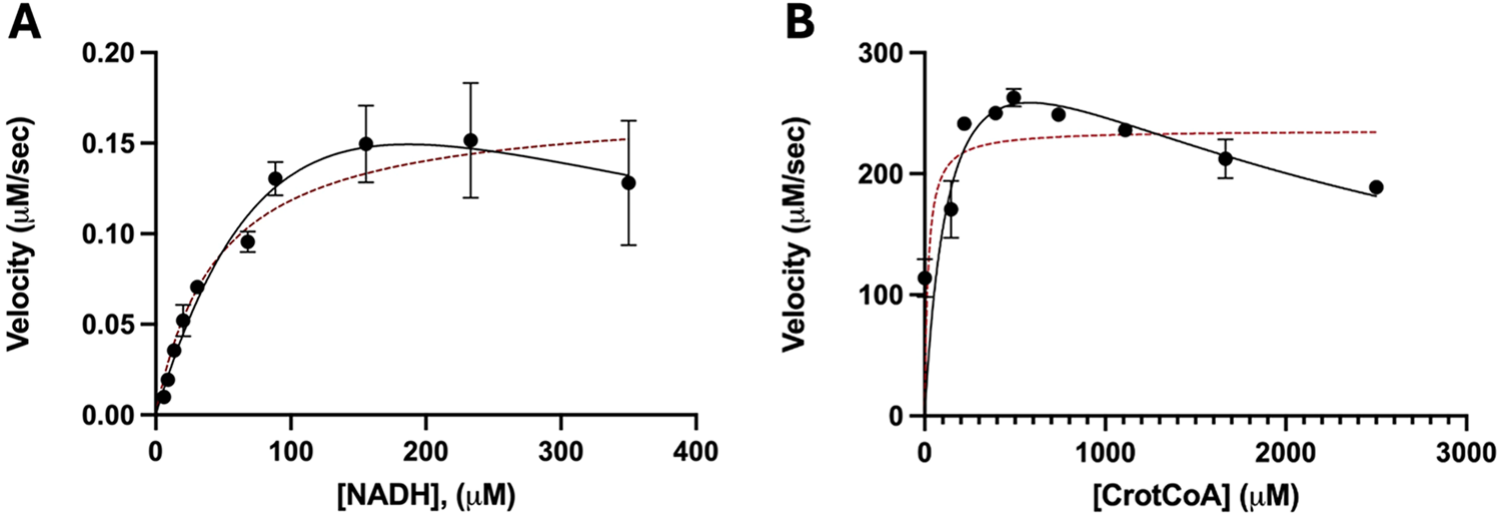
Determination of the apparent *K*_m_ for the *Fn*FabK cofactor (NADH) and the substrate (butenoyl coenzyme-A). The *K*_m_^app^ were determined using a nonlinear regression curve fit of the velocity of *Fn*FabK. (A) The *K*_m_^app^ for NADH was obtained by varying the concentrations of NADH (1.5-fold dilution beginning at 350 μM final concentration) while holding butenoyl-CoA constant at 375 μM. (B) Similarly, the *K*_m_^app^ of butenoyl-CoA was determined by varying its concentration (2.5 mM starting concentration and a 1.5-fold dilution) while holding the NADH concentration at 160 μM. The data was initially fit to the Michaelis-Menten model (red dashed line). However, upon further evaluation, it was determined that the substrate inhibition (solid black line) model proved to be a better fit for the data.

### Benzothiazole Scaffold Derivatives Inhibit FnFabK Activity

The benzothiazole compound, **AG-205** (Figure 2), was previously reported to inhibit *Streptococcus pneumoniae* FabK (*Sp*FabK).^26^ However, it was shown that *S. pneumoniae* can bypass FAS-II inhibition.^27^ Because *Fn*FabK is essential to overall cell growth, **AG-205** was tested against *Fn*FabK in our fluorescence intensity assay to assess the inhibitory activity. No appreciable *Fn*FabK inhibitory activity was observed with **AG-205**. Structural modifications to obtain several benzothiazole analogs (Figure 2) have previously been reported by Norseeda et al, with compound’s activity *vs* *Cd*FabK reported.^23^ To determine the level of inhibitory activity *vs* *Fn*FabK, a series of benzothiazole analogues were tested against purified *Fn*FabK initially at both 100 and 10 μM inhibitor concentrations to determine the percent inhibition. Compounds resulting in >50% inhibition at 10 μM were then assayed in a dose-dependent manner to calculate the IC_50_. A similar fluorescence intensity assay was utilized to counter-screen against *S. aureus* (*Sa*FabI) to determine the degree of selectivity for FabK over FabI.^27^

**Figure 2:**
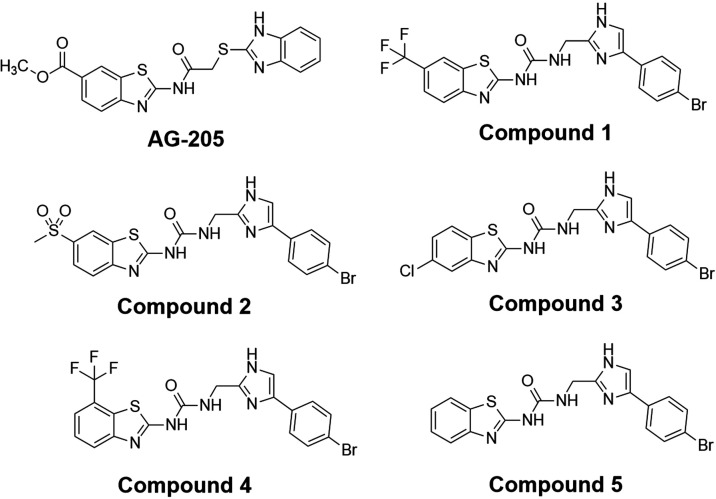
FabK Inhibitors. FabK inhibitors tested in these studies possess a benzothiazole scaffold.^23^

The measured activities showed that the benzothiazole compounds inhibit *Fn*FabK in a dose-dependent manner but lack inhibitory activity against *Sa*FabI. The IC_50_ of the selected compounds ranged from 10 μM with **5** to 0.83 μM with **1** (Table 1). The resulting Hill slope coefficients are not significantly different from 1.0 (Figure S1), suggesting that binding to each active site of the two monomers is independent and occurs without cooperativity. This is also consistent with the previously reported suggestion that, for FabK enzymes, the active sites function independently despite being a functional dimer.^20^ None of the tested compounds showed a significant degree of activity, nor did they show a percent inhibition that is >25% even at 30 μM of inhibitor, when counter-screened against *Sa*FabI (Table 1), suggesting their selectivity for FabK over FabI. To validate the *Sa*FabI inhibitory activity, triclosan, a potent FabI inhibitor, was also tested and showed 99% inhibition at 30 μM.

**Table 1:** Inhibitory Activity and Biophysical Profiles of *F. nucleatum* FabK by Benzothiazole Analogues^a^

compound	*Fn*FabK IC_50_ (μM)	*Fn*FabK *K*_d_ *(*μM*)*	*Fn*FabK % inhibition, 10 μM (%)	*Cd*FabK IC_50_ ^22^ (μM)	*Sa*FabI % inhibition, 30 μM (%)	*Fn*FabK Δ*T*_m_ (°C)	*Fn/Cd* fold-selectivity Index
triclosan	>100				99.9		
1	0.83	2.93	82	0.1	13.5	8.82	8.30
2	2.05	3.98	75	0.43	21.0	8.31	4.77
3	2.03	1.55	77	0.19	9.8	6.59	10.68
4	1.79	3.25	83	2.81	18	8.71	0.64
5	10.64	10.51	50	1.88	ND	4.14	5.66

a IC_50_ and percent inhibitions for compounds used in this study. *C. difficile* FabK IC_50_ values are included for comparison. Triclosan is a FabI inhibitor. Δ*T*_m_, change in melting temperature; ND, not determined. The *Fold-selectivity Index* was computed by taking the ratio of the corresponding IC_50_ values.

### Biophysical Thermal Shift Assay Confirms FnFabK Binding

To confirm on-target binding of the identified *Fn*FabK inhibitors, we optimized a thermal stability assay (TSA) for *Fn*FabK. Specifically, our previously published Thermo-FMN TSA assay was adapted to be performed on a BioRad CFX96 TM. Purified *Fn*FabK was incubated with each compound and heated at a temperature gradient.^22^ The five compounds (Figure 2 and Table 1) have been validated as target inhibitors exhibiting binding, as evident by TSA (Figure 3A). Compounds showed an increase in Δ*T*_m_, with the most potent compound having the highest change in melting temperature being 8.82 °C for **1**, which is also the only compound that showed submicromolar activity (Table 1). Lastly, the binding affinity of **1** was determined using the thermo-FMN assay, by recording dose response measurements that were fitted to the equation developed by Vivoli et al.^28^ The dissociation constant (*K*_d_) was determined for all compounds *vs* *Fn*FabK (Figures 3B and S2). Comparing the binding affinity of compound **1** against *Fn*FabK versus *Cd*FabK reveals a *K*_d_ of 2.98 μM for *Fn*FabK compared to the previously reported *K*_d_ value of 0.304 μM for *Cd*FabK, an approximately 10-fold difference in binding affinity.^19^

**Figure 3:**
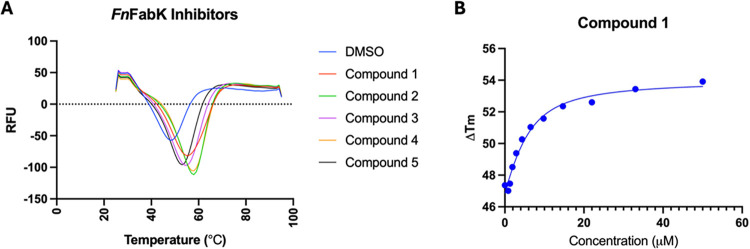
Thermal shift assay. (A) Thermal shift of benzothiazole compounds against *Fn*FabK in the thermo-FMN assay. (B) *K*_d_ for *compound* **1** determined using the thermo-FMN assay and calculated from a dose response curve.

### FnFabK Experimental Structure and Active Site Analysis

To understand the structural basis of the benzothiazole inhibitory activity and improve their current design, we determined the crystal structure of *Fn*FabK bound to **1** at a 2.25 Å resolution. The *Fn*FabK unit cell showed a set of six dimers that were highly packed in the asymmetric unit with a solvent content of ∼36.83% (Figure 4A). The protein cocrystallized in the *P*12_1_1 space group with FMN, the inhibitor, and two sodium ions tightly bound per monomer. The 12 monomers in the unit cell showed similar structures, with minor differences. The high copy number of monomers is therefore advantageous as it is rich in structural information, providing hints on the dynamics of the protein and ligand flexibility. The data reduction and refinement statistics are listed in Table S2.

**Figure 4:**
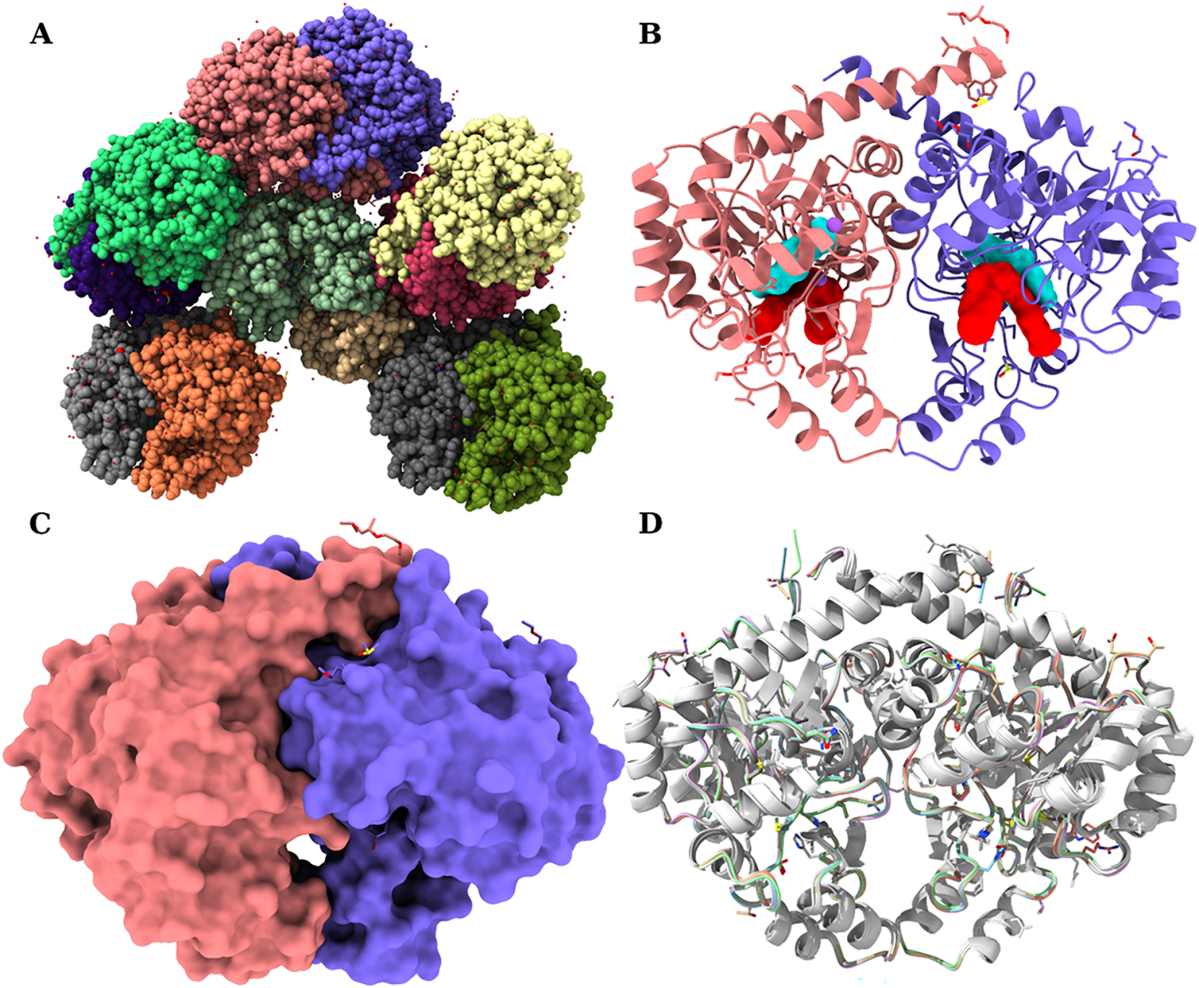
Overall structure of the *Fn*FabK enzyme. (A) The overall structure of FnFabK enzyme asymmetric unit (solved to a 2.25 Å resolution) is composed of six dimers (12 monomers). (B) The structure of a single dimer in ribbon representation (chains A and B in purple and orange, respectively) with the FMN and the bent inhibitor (depicted in cyan and red isosurfaces, respectively), shown π-stacked. (C) An isosurface representation of (B). (D) The aligned asymmetric unit dimers show consistent secondary structure, with most variations found in loops or side chains of a specific and limited set of residues.

FabK is known to function biologically as a dimer.^5,29^ Figure 4B,C, respectively, shows a ribbon and a surface representation of the structure of the *Fn*FabK dimer (chains A and B, depicted in purple and orange, respectively). The alignment of all dimers (Figure 4D) shows a consistent secondary structure across all dimers. Unless otherwise mentioned, throughout the manuscript, we will use chain A as a reference for describing and illustrating the details of the enzyme structure. The *Fn*FabK monomer possesses an overall TIM (triose phosphate isomerase) barrel structural motif, with the characteristic eight β strands forming the central core and eight α helices exterior to the β strands.^18,30,31^ Notably, a large C-terminal domain insertion is present as part of loop 15 (β_8_ → α_8_) composed of an α–α-β-β structural motif. This C-terminal domain has been observed in other FabK structures published and may play a role in cofactor binding and enzyme catalysis.^5,18^ As with other FabK enzymes, the dimers are formed from two monomers that are rotated ∼180° along the longitudinal axis of the dimer, making the two active sites face opposite directions (Figure 4B,C).^5,6,18,24^ The *Fn*FabK active site hosts a flavin mononucleotide (FMN) coenzyme that is essential for its function (Figure S3A).

The inhibitor, **1**, is bound in a hydrophobic binding pocket (Figures S3B and 5A). The pocket is in the proximity of a set of hydrophobic residues that construct the relatively larger, flexible, and hydrophobic active site (Figure 5A,B). These residues include Ala22 (loop 1, β_1_ → α_1_), Gly45 & Gly46 (loop 3, β_2_ → α_2_), Met72 & Leu74 (loop 5, β_3_ → α_3_), Ala97 (loop 7, β_4_ → α_4_), Val117 (loop 9, β_5_ → α_5_), Val146 (loop 11, β_6_ → α_6_), and Leu262 & Met277 (C-term domain, β_8_ → α_8_). Moreover, the inhibitor is in proximity to two histidine residues, His145 & His228, that may play a role in catalysis. As observed in the inhibitor-bound structure of *Cd*FabK,^24^ and reported for the *S. pneumoniae* FabK,^18^ the benzothiazole ring in the ligand makes a π–π stacking interaction with the isoalloxazine ring of FMN (Figure 4B). The ligand phenyl ring stacks against the side chain of His145. In previously reported structures, it has been discussed that the side chain of His145 is likely to play a role in hydride transfer during enzymatic catalysis.^5,18^

**Figure 5:**
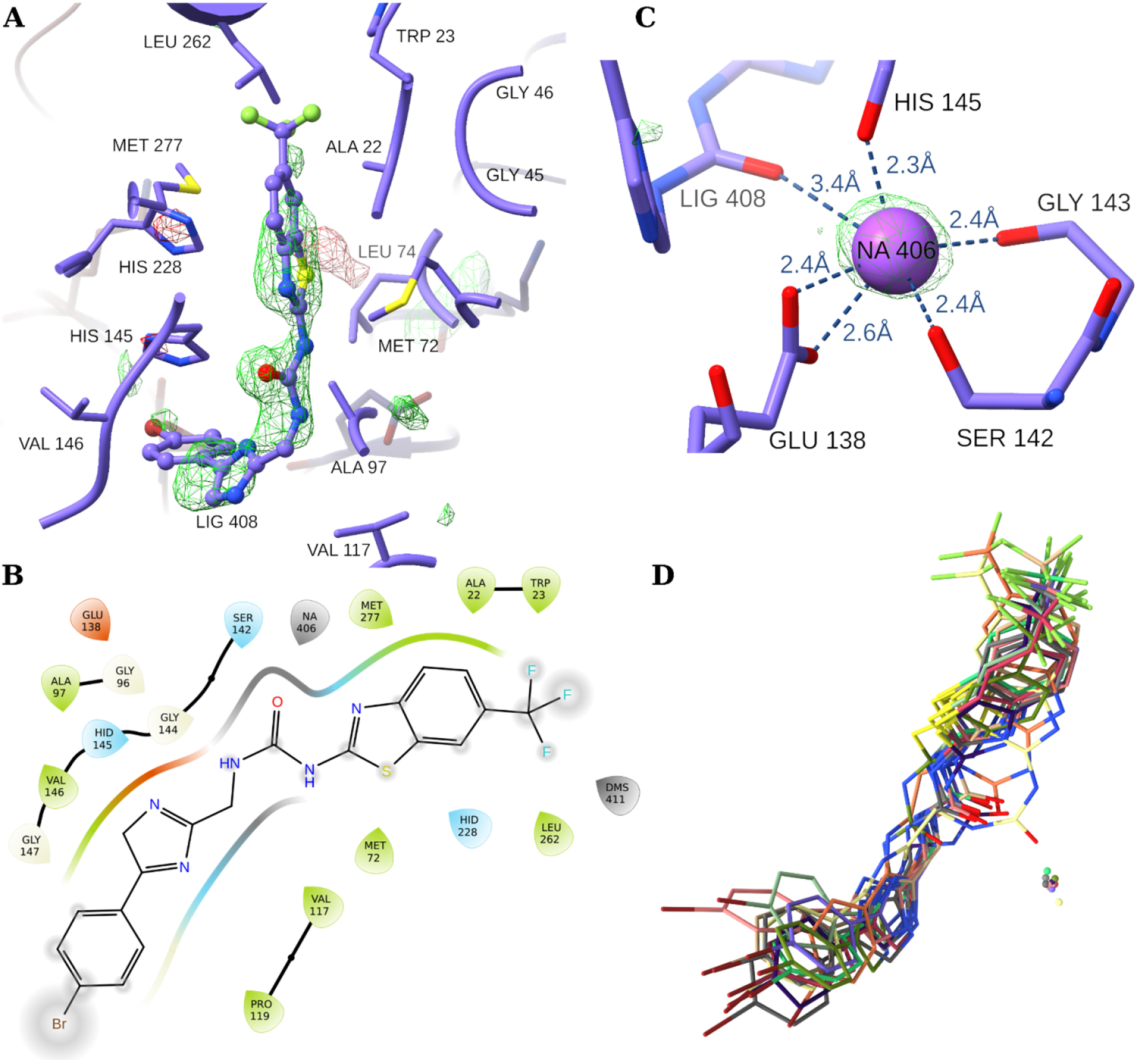
Ligand interactions. (A) Interactions of the ligand (*compound* **1**) with the hydrophobic pocket (FMN is hidden for clarity). The polder (OMIT) map, contoured at 3.5 sigma, is also shown (positive density in green). (B) Ligand interaction diagram. (C) Illustration of the coordination of the sodium ion (Na406) of chain (monomer) (A). The polder (OMIT) map for sodium is shown (contoured at 3.5 sigma). (D) The inhibitor in proximity to the sodium ion Na406 of all the structurally aligned asymmetric unit cell monomers.

As seen in other FabK experimental structures, we observed two sodium ions in each monomer (Figure S3C,D).^5,6,18,24^ One of the ions (Na406) is near the active site and is thought to play a critical role in catalysis (Figure 5C). This ion is bound to a niche structural motif formed as part of loop 11 (β_6_ → α_6_). It is coordinated by both the backbone and side chains of the surrounding residues. The bond lengths between the backbone oxygens of residues His145, Gly143, and Ser142 and the ion are within the range of 2.3–2.4 Å. Further, the two carbonyl oxygens of the Glu138 side chain are at distances of 2.4 and 2.6 Å. In addition to these residues, the inhibitor (residue 408) also contributes to the coordination of this active site sodium ion with a Na–O bond length of 3.4 Å. Similar observations of ligand-Na interactions have been previously reported in docking studies.^23^ To further confirm the interaction between the active site sodium ion and the ligand, we compared the different chains to see whether the same interaction is observed in all asymmetric unit monomers. Since the ligand is flexible, it is expected that, if it is strongly coordinated with the ion, the ligand motion would affect the ion’s position. This is indeed the case as seen in Figure 5D, in which all monomers are aligned. The figure illustrates that a clear correlation is present between the ligand position and orientation and the position of the corresponding sodium ion.

### The Structure–Activity Relationship of Benzothiazole FabK Inhibitors

By analyzing the differences in FabK inhibitory activity of benzothiazole compounds against *Fn*FabK (Table 1), a hypothetical structure–activity relationship (SAR) was formulated. All compounds have identical linker and “tail” groups containing urea, imidazole, and phenyl-bromide groups which are all modifications from the initial **AG-205** benzothiazole compound identified as a *Sp*FabK inhibitor but showing no significant activity against *Fn*FabK. Starting from the unsubstituted benzothiazole scaffold seen in **5**, it is evident that substitutions on the benzothiazole benzene ring increase activity at least 5-fold depending on the site of the substitution. All tolerated substitutions are electron-withdrawing groups, with the group showing the greatest level of activity being a trifluoromethyl group substituted at the C6 position of the benzothiazole ring. When this trifluoromethyl group is substituted at C7 of the same benzothiazole ring in **4**, an ∼2-fold loss in activity is observed. A methyl-sulfonyl group at the C6 position of the benzothiazole ring in **2** resulted in similar levels of activity as seen with **3**, which bears a chlorine substitution at C5. However, both show almost ∼2.5-fold loss in activity compared to **1**, suggesting that an electron-withdrawing substitution on the benzothiazole ring is necessary for the overall activity with the most favorable site of substitution being at C6 with a strongly electron-withdrawing group such as a trifluoromethyl group. We have previously hypothesized that electron-withdrawing substitutions facilitate the inhibitor’s interactions with the active site sodium ion.^23^

### The Active Site Indicates the Presence of Open and Closed Conformational States

To gain insights into the *Fn*FabK enzyme function and ligand dynamics, we superimposed all monomers present in the unit cell and compared the ligand conformations and side chains of the active site residues. The comparisons among all copies show clear indications of ligand flexibility. As indicated by the surface representation in Figure 6A, there is further unoccupied room available in the active site. The hydrophobic pocket can therefore tolerate larger ligands and bulkier substitutions. Since the residues around the ligand are mostly hydrophobic, they tend to adapt their positions responding to the ligand motion (Figure 6B), whereas the ligand itself clearly needs to be able to adopt a bent (L-shaped) conformation to adapt to the active site (Figures 5A,D and 6). Hence, the bent active site favors a flexible ligand. Moreover, the superposition of the ligand poses in the different monomers clearly shows that the ligand binds in a set of diverse configurations that occupy the wider hydrophobic pocket (Figure 6C).

**Figure 6:**
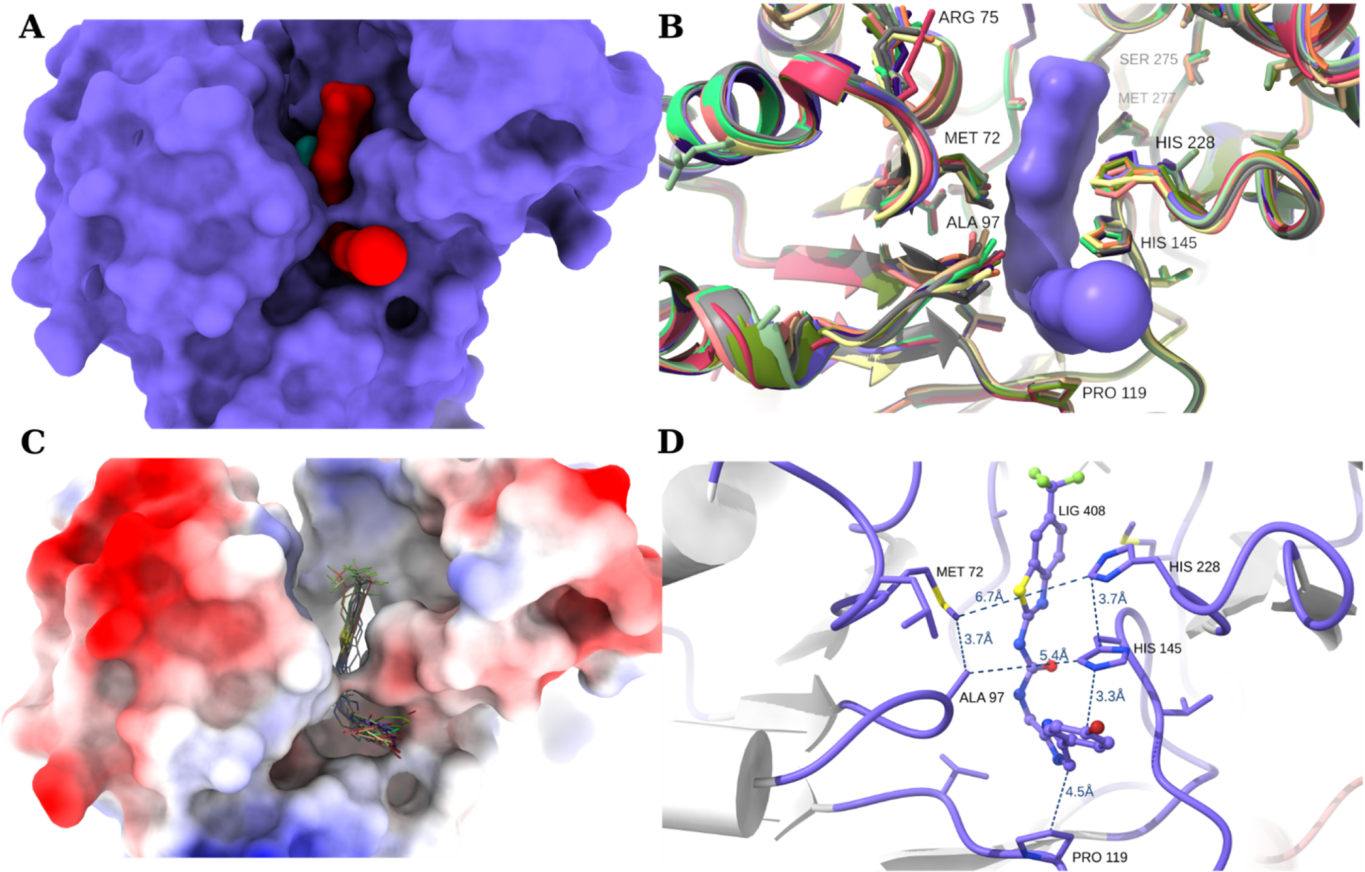
Active site closure and ligand flexibility. (A) Surface representation of the ligand (in red, *compound* **1**, residue 408) in the enzyme (in purple) active site. FMN (in cyan) can be observed behind the ligand. (B) Variations in the side chain conformations of the residues that are in proximity to the ligand. (C) Conformational heterogeneity of ligand in the active site. (D) Interactions that stabilize the active site conformation and binding of the substrate or inhibitor.

The ligand is trapped in the active site (Figure 6A), and for it to enter or leave, the active site needs to open. This suggests the presence of open and closed states, and that a conformational switch may take place for the substrate or inhibitor to enter or leave the active site. This is supported by changes in the side chain conformations of residues near the ligand, which corresponds to Leu74 (loop 5), Ala97 & Asn99 (loop 7), His145 (loop 11), and His228 (C-term domain, loop 15) in *Fn*FabK, between the *S. pneumoniae* apo and bound states (PDB ID 2Z6I and 2Z6J, respectively). These side chain changes were accompanied by a slight adaptation of the loops having these residues. Moreover, the side chains of the residues in the active site, in proximity to the ligand, exhibit a degree of conformational heterogeneity (Figure 6B) between the different copies of the enzyme monomers, indicating a certain degree of active site flexibility, which is consistent with our hypothesis that open and closed conformations may exist. Figure 6C shows that the ligand adopts slightly different conformations, all of which have a characteristic bent (L-shaped) conformer. It appears that the active site can adopt a certain degree of conformational heterogeneity. Despite this flexibility, the active site is stabilized by a set of hydrophobic interactions, including a series of π-stacking along the active site between the aromatic rings of His228, His145, the inhibitor imidazole ring, and Pro119 (Figure 6D). These residues may also be stabilized by hydrophobic interactions across the active site entrance with residues Met72 and Ala97. Collectively, these interactions provide a means of zipping the active site and stabilizing the substrate or inhibitor once they enter the active site and, hence, retain the enzyme closed state until the reaction is complete. Thus, for the ligand to enter the binding pocket, the active site needs to be opened by the local movement of the side chains and possibly the backbone atoms of loops surrounding the ligand.

## Discussion

### Structural Basis for Inhibitor Selectivity

Interestingly, when **1** was tested against *Clostridioides difficile* (*Cd*FabK), it was 8-fold better with an IC_50_ of 0.10 μM, compared to 0.83 μM for *Fn*FabK. This suggests that some degree of bacterial selectivity can be achieved, even among organisms that are FabK expressers. The comparison of the structure of *Cd*FabK with that of *Fn*FabK determined in this study (Figure S4 and alignment in Figure S5) reveals that Met72 in *Cd*FabK is substituted by the shorter leucine residue (Leu74) in *Fn*FabK. In *Cd*FabK, Met72 stretches out to interact with His143 (His145 in *Fn*FabK). One result of the latter is strengthening the interaction of two loops near the active site, hence tightening the active site and effectively reducing the ligand motion. Thus, the substitution with a shorter residue in *Fn*FabK above ligand bromine atom makes this region of the active site wider and hence allows the ligand tail to move freely, resulting in flexible poses. A second noticeable difference between the two structures is that residue Glu146 in the *Cd*FabK active site is replaced by a polar threonine (Thr148) in *Fn*FabK. Such substitution leads to the loss of the strong interaction between Arg215-Glu146 observed in *Cd*FabK. The latter two interacting loops contribute to the zipping of the active site and hence strengthen the overall interactions of the ligand with the neighboring hydrophobic residues. Further, the presence of a longer charged residue in *Cd*FabK (Glu146) exerts an electrostatic pressure that constrains the motion of the hydrophobic tail of the bound ligand. This effect is diminished for the shorter polar residue (Thr148) in *Fn*FabK.

The overall effect of these residue substitutions is an entropic gain for *Fn*FabK but is also associated with a decrease in overall interactions (enthalpic loss) of the active site hydrophobic residues with the ligand. These findings partly explain the differences in the observed IC_50_ for **1** between *Cd*FabK and *Fn*FabK and provide insights into the design of novel inhibitors that may achieve more species specificity. Moreover, molecular docking of compound **1** to *Cd*FabK (Figure S6) suggests that the amino groups in the ligand linker as well as the nitrogen in the benzothiazole ring interact more strongly with the His143 (compared to their interaction with the corresponding His145 in *Fn*FabK), whereas the ligand linker carbonyl group coordinates the sodium ion in *Fn*FabK.

### The Shape of the Active Site Is a Determinant for Ligand Selectivity

Cell membranes have lipid compositions that can vary significantly among bacterial species, with respect to chain length, unsaturation, and chain branching.^32^ FabK enzymes reduce a trans-2-enoyl double bond during the bacterial fatty acid elongation cycle. These enzymes must accommodate fatty acid substrates of varying lengths and structures, including branched-chain fatty acids and unsaturated fatty acids, depending on the lipid membrane requirements of the specific organism. We therefore hypothesize that the overall shapes of the active sites have evolved to better accommodate the type of fatty acid that is required for each specific bacterial species and that this subsequently influences the variations in the binding affinity and potency of inhibitor compounds observed in FabK enzymes from the different species. Thus, the presence of specific classes of lipids in certain species may influence the corresponding shape of the substrate binding sites. Structural differences between the fatty acid substrates of these enzymes may provide insights on the shape of the substrate binding pockets, which may be leveraged toward inhibitor design. For example, Sohlenkamp and Geiger pointed out that Firmicutes falling within the Clostridia class and *Streptococcus* genus contain or utilize branched-chain fatty acids.^32^ Branched-chain fatty acids have bulkier tails compared to those of unbranched fatty acids. Further, it has been shown that certain bacterial species are able to change their lipid membrane composition in response to the environment. For instance, an increase in the proportion of unsaturated fatty acids was reported in *S. pneumoniae* in certain phenotypes resulting in an increase the membrane fluidity.^33^ Unsaturated fatty acid chains, with a cis-double bond, are less flexible compared to saturated ones and hence may be influenced by the width of the active site. Ultimately, the combination of different types of lipids, their proportions, chain lengths, and the molecular geometry of individual lipid molecules are factors that affect the overall membrane characteristics such as fluidity, curvature, density, and packing.^34^

The known membrane lipid composition of selected bacterial species of interest is listed in Table S3. Figure 7A–C shows the active site shapes for *Fn*FabK, *Cd*FabK, and *Sp*FabK, respectively. The presence of branched (bulkier) chain fatty acids in *C. difficile* and *S. pneumoniae* (Table S3) may explain the need for wider substrate binding sites observed in these species compared to *F. nucleatum*, which tends to use less bulky unsaturated fatty acids (Table S3). Indeed, the active site of the *Fn*FabK (Figure 7A) has a smaller width (in certain parts of the active site) than the corresponding site of *Cd*FabK (Figure 7B) and *Sp*FabK (Figure 7C). Therefore, the shape of the active site in its closed state may be an indicator of the preferred fatty acid substrate type. Further studies are needed to confirm such a hypothesis. Despite this essential and delicate selectivity, the active site of bacterial species also needs to be able to tolerate the growing fatty acid chain in the FAS-II cycle with its varying lengths and structures. Indeed, both active site plasticity and ligand flexibility are evidence that enzymes can tolerate such a range of lipid substrates. The features described and the unique active site structural features of FabK enzymes from different species present further opportunities to design species-selective inhibitors, even within FabK-expressing organisms.

**Figure 7:**
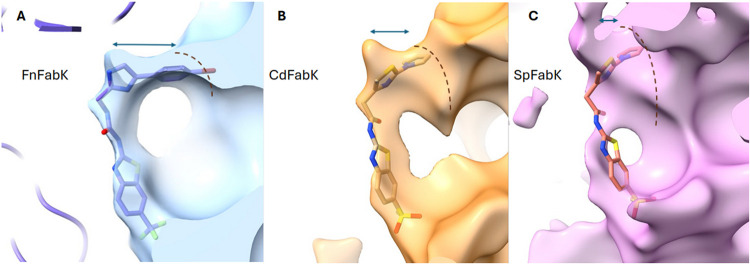
FabK Active Site Morphology. A comparison of the active site morphology in FabK enzymes of three bacterial species; (A) *F. nucleatum* (PDB ID 9PL0, this study), (B) *C. difficile* (PDB ID 7L00), and (C) *S. pneumoniae* (PDB ID 2Z6I). The three models were aligned to identify the ligand binding region. The shape of the active site was extracted based on the PDB models as described in the Methods section. The resulting maps illustrate the characteristic size and shape of the space available for the ligand-based PDB model. The *Fn*FabK enzyme model along with its inhibitor is shown in (A), and only the ligand (residue XCJ in PDB ID 7L00) is shown in (B) and (C) for clarity.

## Conclusion

The benzothiazole compounds studied herein possessed low to submicromolar *Fn*FabK inhibitory activity with no appreciable inhibition of *Sa*FabI, demonstrating FabK selectivity. Additional biophysical analyses confirmed FabK target engagement. The noted differences in compound activity among FabK-expressing organisms indicate that species-specific selectivity is possible, even within this group. The structure of the FabK enzyme from *F. nucleatum* offers valuable insights into the design of new inhibitors that leverage structural variations in FabK binding pockets to specifically target different bacterial species. Moreover, the high copy number of monomers in the unit cell gave novel insights into the enzyme conformational states. The comparison between the ligand conformers in the different monomers suggests at least two distinct enzyme states with open and closed active sites. Moreover, the comparison between the FabK structures from the different bacterial species not only explained the underlying molecular mechanism of the selective ligand inhibition but also unveiled a potential structural basis for distinct natural substrate preferences among the studied bacterial species.

## Materials and Methods

### Expression of FnFabK and SaFabI

The *fabK* and *fabI* genes from *F. nucleatum* and *S. aureus* were cloned within the NdeI/*Bam*HI site of pET15b. Both constructs were then codon-optimized for *Escherichia coli*, synthesized, and cloned by GeneWiz from Azenta Life Sciences. The proteins were overexpressed in *E. coli* BL21-Gold (DE3) by inoculating 500 mL of Terrific Broth (TB) having a 100 μg/mL ampicillin with 5 mL starter cultures of Luria–Bertani (LB) broth, and the cells were grown at 37 °C while being shaken at 250 rpm. Cells were grown to an OD_600_ of ∼0.6, and protein expression of *Fn*FabK was induced by supplementing the media with 0.1 mM isopropyl β-d-1-thiogalactopyranoside (IPTG) and 0.5 mM flavin mononucleotide (FMN) for enhanced expression. Following induction, the temperature was lowered to 18 °C and the cells were grown, shaking, for 18 additional hours at 220 rpm. The *Sa*FabI protein was similarly overexpressed in *E. coli* BL21-Gold (DE3) by inoculating 500 mL of Terrific Broth (TB) with 100 μg/mL ampicillin with 5 mL starter cultures of Luria–Bertani (LB) and grown at 37 °C while shaken at 250 rpm. Cells were grown to an OD_600_ of ∼0.6, and the protein expression was induced with 1 mM IPTG. Following induction, the temperature was maintained at 37 °C and the culture was grown for 4 additional hours while shaking at 220 rpm. Cells were then harvested by centrifugation at 10,000 rpm for 15 min at 4 °C for immediate use or stored in −80 °C prior to subsequent purification.

### Purification of FnFabK and SaFabI

The *Fn*FabK pellets were resuspended for purification in lysis buffer containing 50 mM Tris pH 8.0, 100 mM NH_4_Cl, 10% v/v glycerol, 100 μM FMN, 5 mM imidazole, 0.5 mg/mL lysozyme, 0.5% Triton-X 100, 5 mM MgCl_2_, 25 mM sucrose, 2 mM DTT, 1 mg/mL DNase, and 1.5 mL protease inhibitor cocktail per 100 mL. For every gram of cells grown, 10 to 15 mL of lysis buffer was utilized. Cells were lysed on a stir plate at 4 °C for 1 h. Following cell lysis, the suspension was sonicated at 50% amplitude for a total of 8 min “on time” using a cycle of 8 s on and 24 s off while sample remained on ice. Lysate was centrifuged at 18,000 rpm at 4 °C for 15 min, after which the supernatant was passed through a 0.45 μm filter. The first step of purification took place via affinity chromatography on a His Trap HP column (Cytiva Life Sciences). The binding buffer contained 50 mM Tris pH 8.0, 100 mM NH_4_Cl, 10% v/v glycerol, 100 μM FMN, 5 mM imidazole, and 2 mM DTT. The elution buffer contained the same components as the latter with increased imidazole concentration of 250 mM. The eluted protein was further purified by gel filtration on Superdex 200 PG (Cytiva Life Sciences) using the running buffer containing 50 mM HEPES pH 8.0, 300 mM NH_4_Cl, 10% v/v glycerol, 2 mM DTT, and 100 μM FMN. Similarly, the *Sa*FabI pellets were resuspended for purification in lysis buffer containing 50 mM Tris pH 8.0, 500 mM NaCl, 5% v/v glycerol, 10 mM imidazole, 0.5 mg/mL lysozyme, 0.5% Triton-X 100, 5 mM MgCl_2_, 1 mM DTT, 1 mg/100 mL DNase, and 1.5 mL protease inhibitor cocktail per 100 mL. Affinity chromatography buffer contained 50 mM Tris pH 8.0, 500 mM NaCl, 5% v/v glycerol, 10 mM imidazole, and 1 mM DTT for binding buffer and elution buffer contained the same components with increased imidazole concentration of 500 mM. The gel filtration running buffer contained 50 mM Tris HCl pH 8.0, 100 mM NaCl, 5% v/v glycerol, and 1 mM DTT. After purification, concentrated proteins were stored at −80 °C in 30% v/v glycerol for utilization in either *Fn*FabK or *Sa*FabI FI assay.

### Differential Scanning Fluorimetry (Thermal Shift Assay)

All compounds were initially dissolved in 100% DMSO at a final concentration of 10 mM and then further diluted in DMSO to the required concentrations. The thermal shift assay was conducted as follows. Reactions were started at 23 °C and the temperature was increased by 0.03 °C/s up to a final temperature of 65 °C. Purified *Fn*FabK at a concentration of 5 μM was incubated for 10 min with each compound at 50 μM and heated at a temperature gradient. DMSO was used as a negative control. The melting temperatures were measured using a BioRad CFX96 with the FAM filter with excitation and emission wavelengths of 492 and 516 nm, respectively. Additionally, the thermodynamic dissociation constant was determined for all compounds in a dose-dependent manner, as previously published using the Thermo-FMN assay.^22,28^

### Biochemical Assay for FnFabK

The *Fn*FabK assay was conducted by the following protocol. Reactions were carried out at 25 °C in assay buffer (100 mM HEPES pH 8.0, 500 mM NH_4_Cl, 10% v/v glycerol) with 150 μM butenoyl-CoA and 160 μM NADH. FabK enzyme was diluted using 2.5 mg/mL γ globulin in the assay buffer to a working stock of 0.6 μM, for a final concentration of 30 nM, and was incubated with compound at 100 and 10 μM. Incubation lasted for a total of 10 min before the addition of butenoyl-CoA substrate, and the reaction was started with the addition of NADH for a final assay volume of 100 μL. The oxidation of NADH to NAD^+^ was measured by tracking fluorescence (340/460 nm) with a Biotek Synergy H1 microplate reader in 15 s intervals for a total of 10 min to monitor the rate of reaction. Reactions were conducted in Greiner Bio-One 384-Well μClear Bottom Polystyrene Microplates. Compounds yielding higher than 50% inhibition at 10 μM were then analyzed in dose response ranging from 100 to 0 μM. For IC_50_ calculations, linear slopes were measured for the first 5 min and used to determine the reaction rates. Measurements were conducted in triplicate, and IC_50_ values were calculated via GraphPad Prism 9.1.2 using four-parameter logistic (Hill) curve analysis.^35^

### Biochemical Assay for SaFabI

The *Sa*FabI assay was conducted as follows. Reactions were carried out at 25 °C in the assay buffer (50 mM MES pH 5.5, 100 mM NaCl). FabI enzyme was diluted in assay buffer to a final concentration of 200 nM and was incubated with compounds at 30 μM. The known FabI inhibitor, Triclosan, was the positive control, and DMSO served as the negative control. Incubation lasted for a total of 20 min before the addition of NADPH cofactor at a final concentration of 300 μM, and the reaction was started with the addition of Butenoyl-CoA at 4 mM final concentration for a final assay volume of 100 μL. The oxidation of NADPH to NADP^+^ was measured by tracking fluorescence (340 nm/460 nm) with a Biotek Synergy H1 microplate reader in 15 s intervals for a total of 10 min to monitor the rate of reaction. Reaction was conducted in Greiner Bio-One 384-Well μClear Bottom Polystyrene Microplates. The percent inhibition of each compound was calculated on the basis of the resulting velocity.

### Crystallization and Structure Determination of FnFabK

Protein crystals were obtained by using the hanging drop vapor diffusion method. Crystals grew in drops containing a 2:1 ratio of the protein solution to precipitant, respectively. The protein solution (12.6 mg/mL) used was buffer exchanged into 10 mM HEPES pH 8.0 and 50 mM NH_4_Cl and was then incubated with **1** at a final concentration of 8.3 μM (final DMSO concentration was 0.2%), for 1 h at room temperature. Crystals grew best when using solutions having 25% Jeffamine ED-2001 (pH 7.0) and 0.1 M sodium citrate tribasic (pH 5.0) as a precipitant. Crystals grew within 24 h at 18 °C. Before cryoprotection using a 70% sucrose solution, the crystals were briefly soaked with 10 mM solution of **1**.

### Data Processing and FnFabK Structure Solution

X-ray diffraction data was collected at the NYX beamline, National Synchrotron Light Source II, Brookhaven National Laboratory, Upton, NY. Data reduction was performed using Dials^38^ (through the xia2 pipeline).^36^ Data sets from two crystals were combined using xia2.multiplex.^37^ The structure was solved by molecular replacement using the dimer obtained from the model PDB ID 7L00. The molecular replacement search used the Phaser software in the Phenix package. In the latter, the initial partial solution was obtained, which had gaps in the unit cell that had the shape of the dimer was used for subsequent search after modifying the expected solvent content parameters in the Phaser. This had helped to overcome noncrystallographic symmetry (NCS) and find a phasing solution that gave reasonable *R*-factors upon refinement. Model Building was carried out in COOT,^38,39^ and refinement was performed with torsion angles NCS constraints using Phenix.refine.^40,41^ Figures were generated using UCSF ChimeraX.^42–44^ The sequence alignment was performed using MUSCLE program,^45^ and alignment figures were generated using JalView.^46^

### Molecular Modeling and Structure Analysis

To determine the shape of the active site of the FabK enzymes, we constructed homogeneous density maps of the protein regions that are expected to be occupied by the solvent. To construct such density, we computed an electron density map of the protein model after removing all solvents and ligands except for FMN using the GEMMI library.^47^ We then generated a mask for the regions occupied by the protein based on a threshold. Areas that had more than 10^–5^ electrons were considered occupied by the protein and were set to zero. The remaining regions were considered to represent the solvent, and their density values were set to unity. Note that this same threshold is used in the calculation of the electron density by Fast Fourier transform (FFT) and hence guarantees that the mask would hide the protein region, and the resulting density maps depict the solvent regions outside the protein atomic radii.

Molecular docking of compound **1** to *Cd*FabK (PDB ID 7l00) was performed using Maestro (version 14.5.131) of the Schrödinger suite (released 2025–3). Briefly, the protein and ligand were prepared using Protein Preparation Workflow and LigPrep applications, respectively. Then, Glide^48^ was to dock the compound using standard precision settings.

## Supplementary Material



## Notes

Biochemical analysis of benzothiazole scaffolds and statistical analysis; *K*_D_ determinations and statistical analyses; electron density and polder maps for *Fn*FabK costructure; comparative analysis of FabK structure from *Cd* and *Fn;* sequence alignment of *Cd*FabK and *Fn*FabK; predicted binding orientation of compound 1 to *Cd*FabK; purity analysis of compounds via LC-MS; common commensal and pathogen organisms ENR expression; common oral pathogens ENR expression; data collection and refinement statistics for *Fn*FabK costructure; and membrane lipids composition of FabK expression species in this study (PDF)
