# Supplementary material for: Structural and Biochemical Characterization of Fusobacterium nucleatum Enoyl-ACP Reductase II (FabK) Reveals the Basis for Bacterial Species-Specific Inhibition
Source: ACS Bio Med Chem Au. 2025 Nov 19;6(1):44–55. doi: 10.1021/acsbiomedchemau.5c00199 (PMC12921513; doi:10.1021/acsbiomedchemau.5c00199)
Supplement: Supplementary file 1 [file bg5c00199_si_001.pdf]

Supporting Information

Structural and biochemical characterization of *F. nucleatum* enoyl-ACP reductase II (FabK) reveals the basis for bacterial species-specific inhibition.

Kristiana Avad[1,§], Osama Alaidi[1,§], Destiny Okpomo[1], Fahad Bin Aziz Pavel[1], Darcy Doran[1], Madeline Matheson[1], Dianqing Sun[3], Julian Hurdle[2], Kirk E Hevener[1,*]

[1] Department of Pharmaceutical Sciences, College of Pharmacy, University of Tennessee Health Science Center, Memphis, TN, 38163, USA.

[2] Department of Translational Medical Sciences, Center for Inflammatory and Infectious Diseases, Institute of Biosciences and Technology, Texas A&M Health Science Center, Houston, TX, 77030, USA.

[3] Department of Pharmaceutical Sciences, The Daniel K. Inouye College of Pharmacy, University of Hawaii at Hilo, Hilo, HI, 96720, USA.

[§]KA and OA contributed equally to this work.

***Corresponding Author:** Kirk E. Hevener, khevener@uthsc.edu.

Supporting Figures

Figure S1. Benzothiazole IC_{50} plots and statistical analyses.

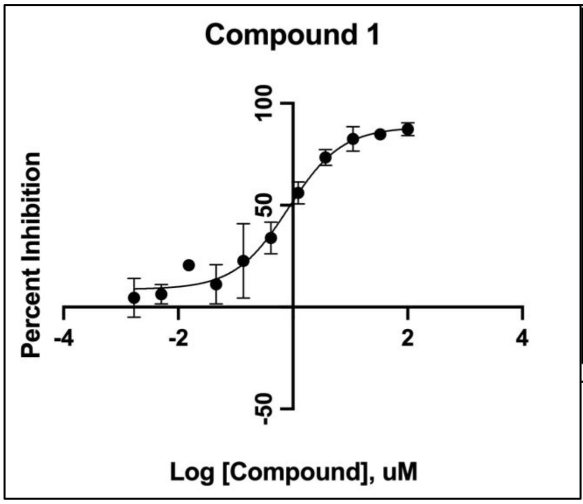

Compound 1	
IC_{50}	0.8305
Hill Slope	0.9325
Degree of Freedom	29
R squared	0.9477
Sy.x	7.945

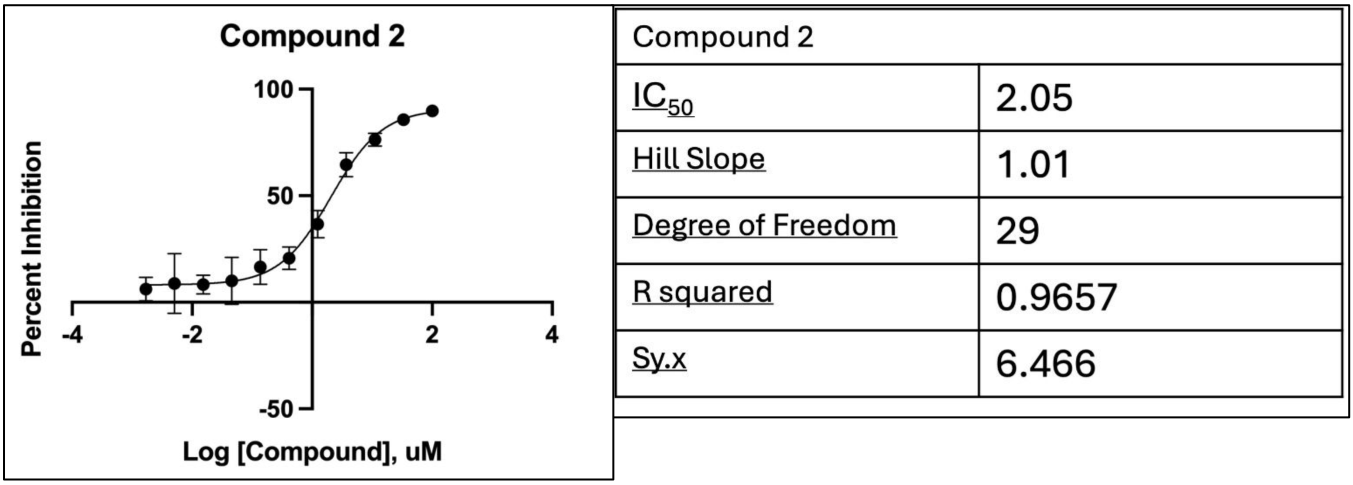

Compound 2	
IC_{50}	2.05
Hill Slope	1.01
Degree of Freedom	29
R squared	0.9657
Sy.x	6.466

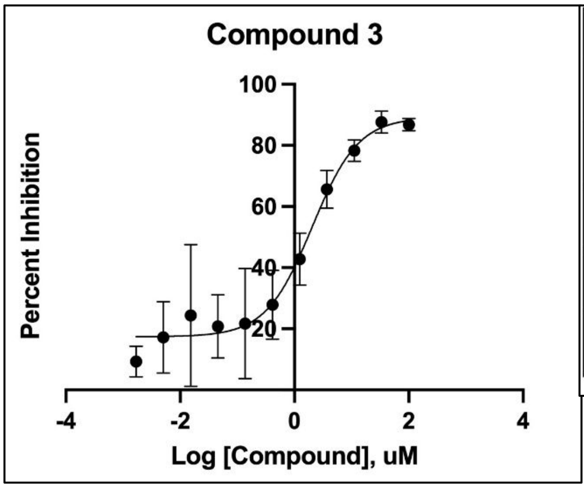

Compound 3	
IC50	2.036
Hill Slope	1.07
Degree of Freedom	29
R squared	0.8937
Sy.x	10.44

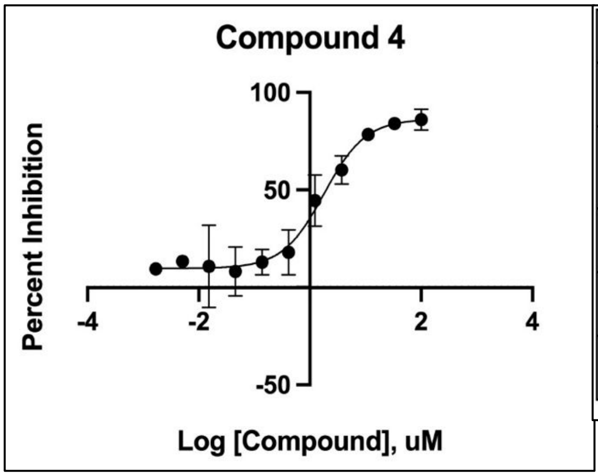

Compound 4	
IC50	1.79
Hill Slope	1.16
Degree of Freedom	29
R squared	0.9322
Sy.x	8.936

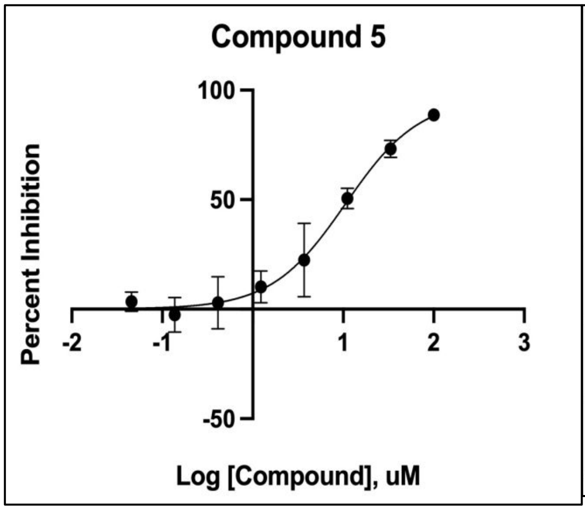

Compound 5	
IC50	10.64
Hill Slope	1.063
Degree of Freedom	20
R squared	0.9530
Sy.x	7.993

Figure S2. K_d Determination from Thermo-FMN Assay and statical analysis.

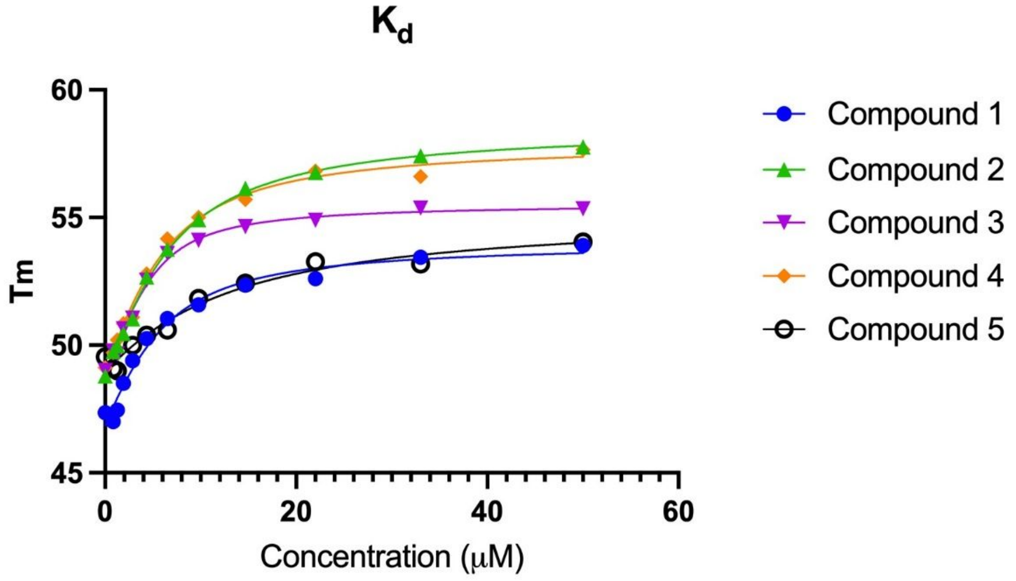

Compound 1	
K_d	2.98
Degree of Freedom	9
R squared	0.9788
Sy.x	0.3939

Compound 2	
K_d	3.98
Degree of Freedom	9
R squared	0.9974
Sy.x	0.1837

Compound 3	
K_d	1.556
Degree of Freedom	9
R squared	0.9925
Sy.x	0.2304

Compound 4	
K_d	3.256
Degree of Freedom	9
R squared	0.9914
Sy.x	0.3143

Compound 5	
K_d	10.51
Degree of Freedom	9
R squared	0.9681
Sy.x	0.3617

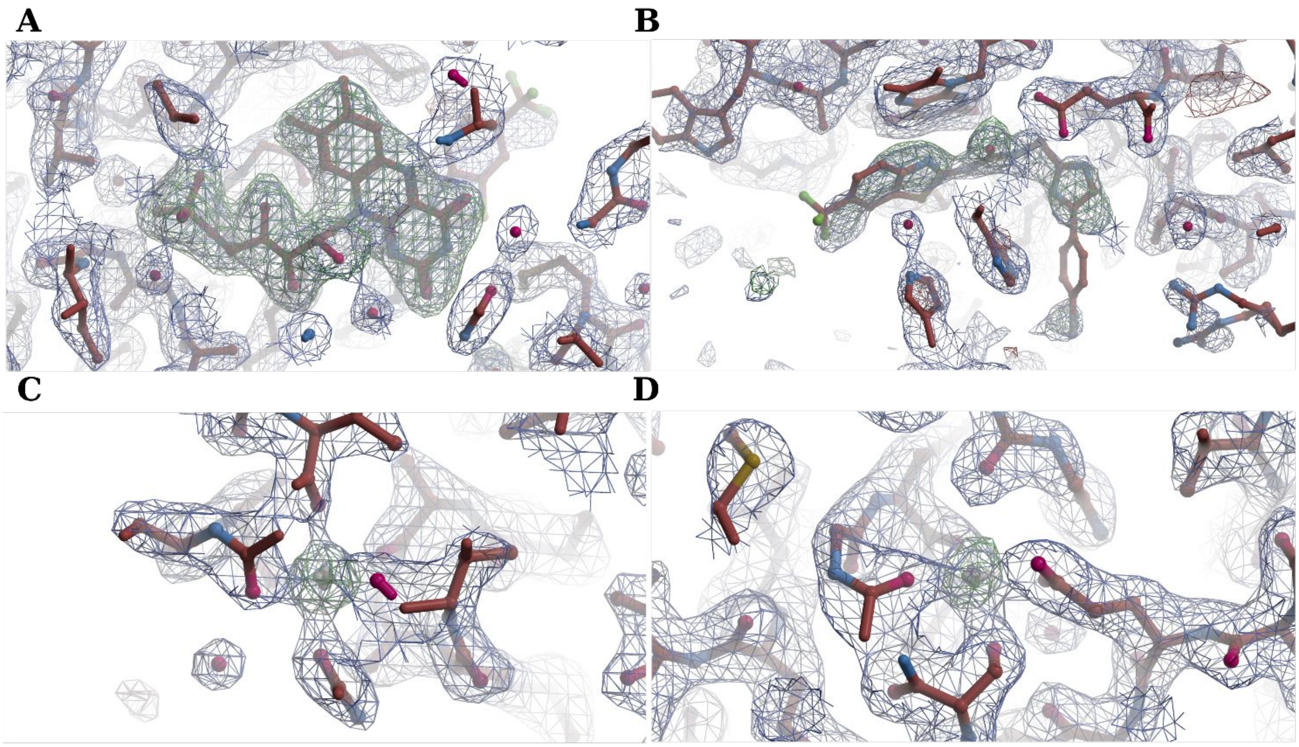

Figure S3. Electron density and polder maps. The figure illustrates the $2F_o$-F_c and the polder (OMIT) maps superimposed for the (**A**) FMN (contoured at 1.0σ and 3.0σ , respectively) (**B**) inhibitor (*compound 1*) (**C**) first sodium ion (Na405) and (**D**) second sodium ion (Na406). The electron density and polder (positive density is depicted in green) maps in panels (**C** and **D**) are contoured at 1.0σ and 3.5σ, respectively. Due to the lower occupancy of the ligand, and for clarity, the latter two maps shown in panel **B** are contoured at 0.8σ and 3.0σ, respectively.

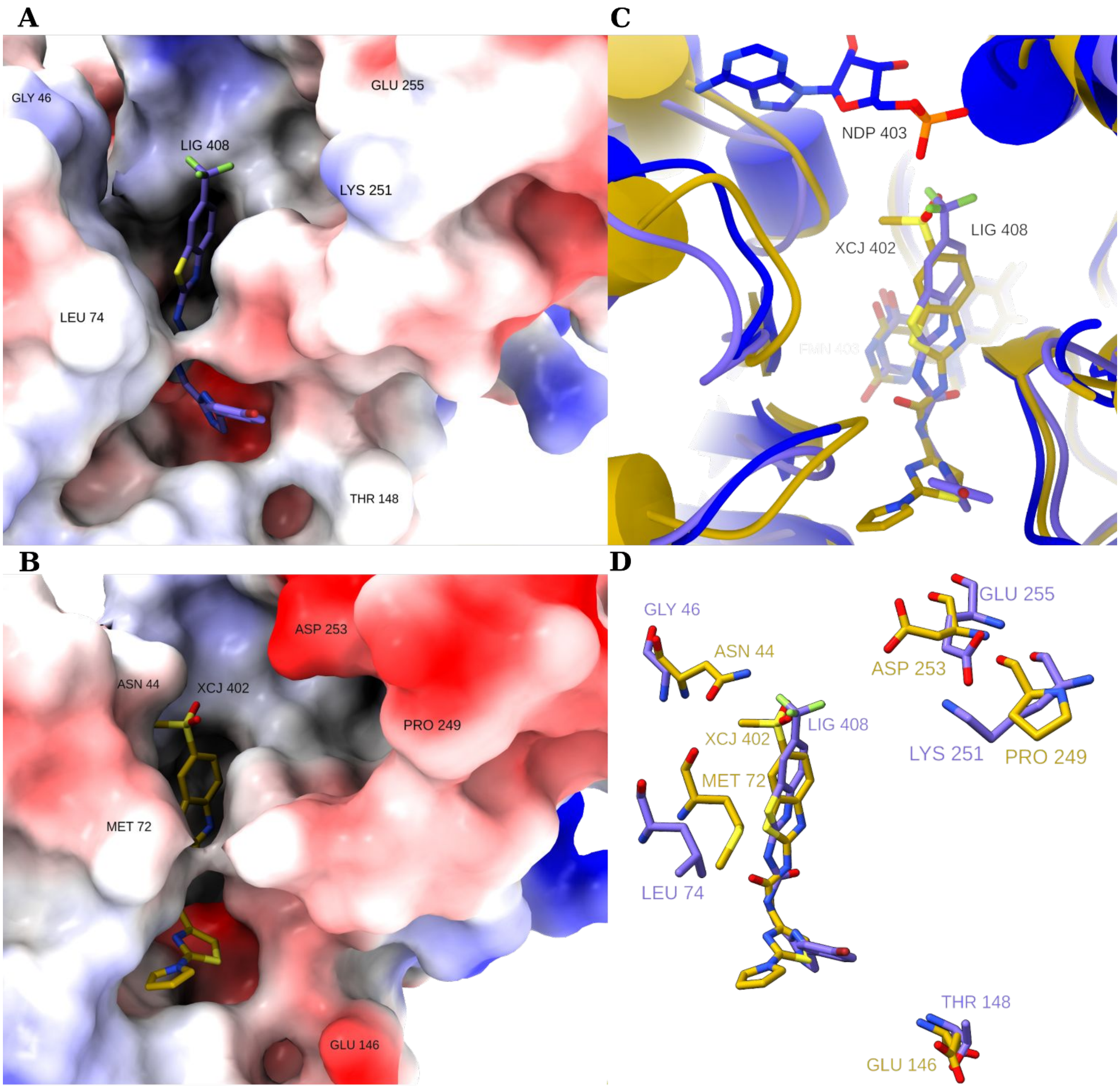

Figure S4. Comparing FabK from different species. A comparison between (**A**) *Fn*FabK-*compound1* and (**B**) *Cd*FabK- XCJ (PDB ID 7L00), showing the corresponding electrostatic isosurfaces. (**C**) The superposition of *Fn*Fabk, *Pg*FabK and *Cd*Fabk structures confirms the position of NADH relative to the ligand. (**D**) Models shown in panels **A** and **B** superimposed with the isosurface hidden to illustrate the key residue differences between the FabK enzyme active sites in the two species.

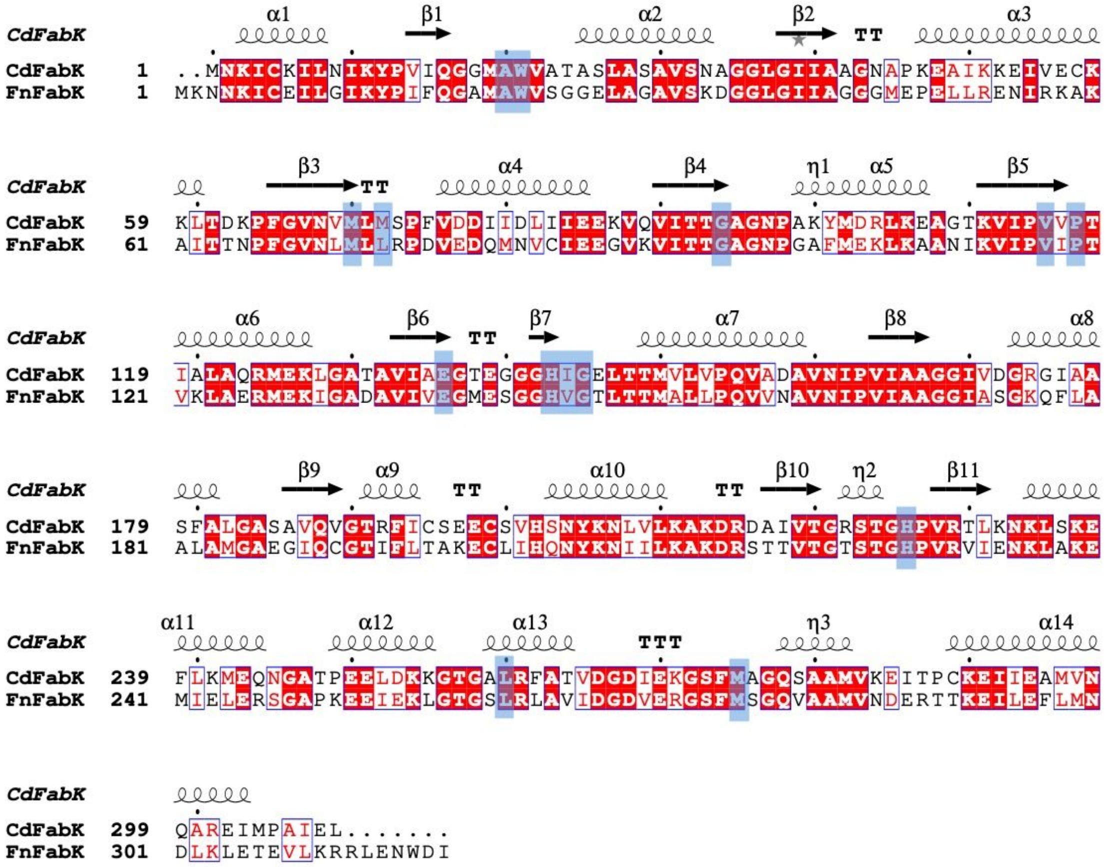

Figure S5. *Cd*FabK & *Fn*FabK sequence alignment. Active site residues falling within 4 Å of the bound inhibitor are highlighted.

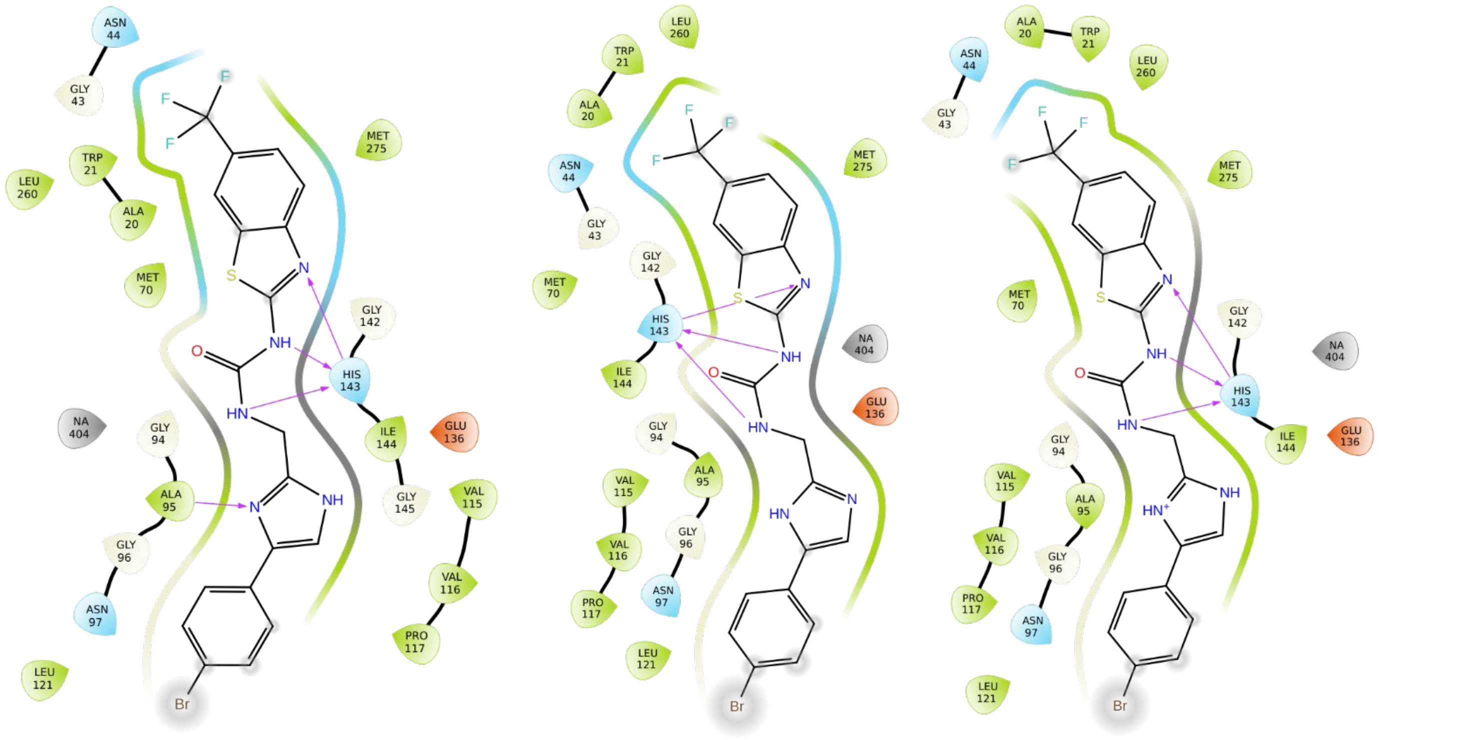

Figure S6. Predicted binding of compound 1 to *Cd*FabK. The docked binding poses of compound 1 are shown, with the lowest energy on the left and the higher energy on the right.

Compound 1

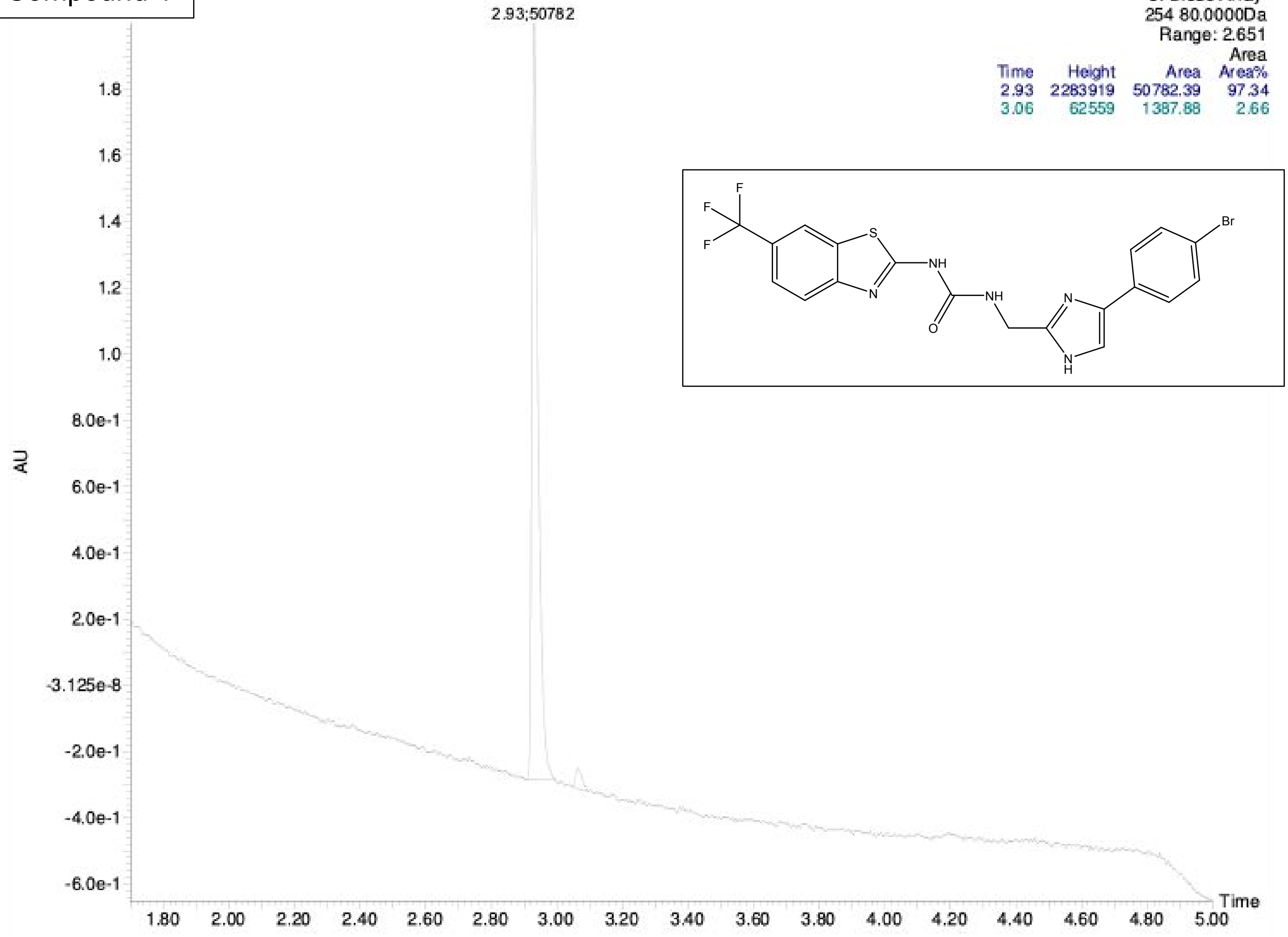

2.93;50782
3: Diode Array
254 80.0000Da
Range: 2.651
Area
Time Height Area Area%
2.93 2283919 50782.39 97.34
3.06 62559 1387.88 2.66
F
F
F
S
N
NH
O
NH
N
N
H
Br
AU
1.8
1.6
1.4
1.2
1.0
8.0e-1
6.0e-1
4.0e-1
2.0e-1
-3.125e-8
-2.0e-1
-4.0e-1
-6.0e-1
Time
1.80 2.00 2.20 2.40 2.60 2.80 3.00 3.20 3.40 3.60 3.80 4.00 4.20 4.40 4.60 4.80 5.00

Compound 2

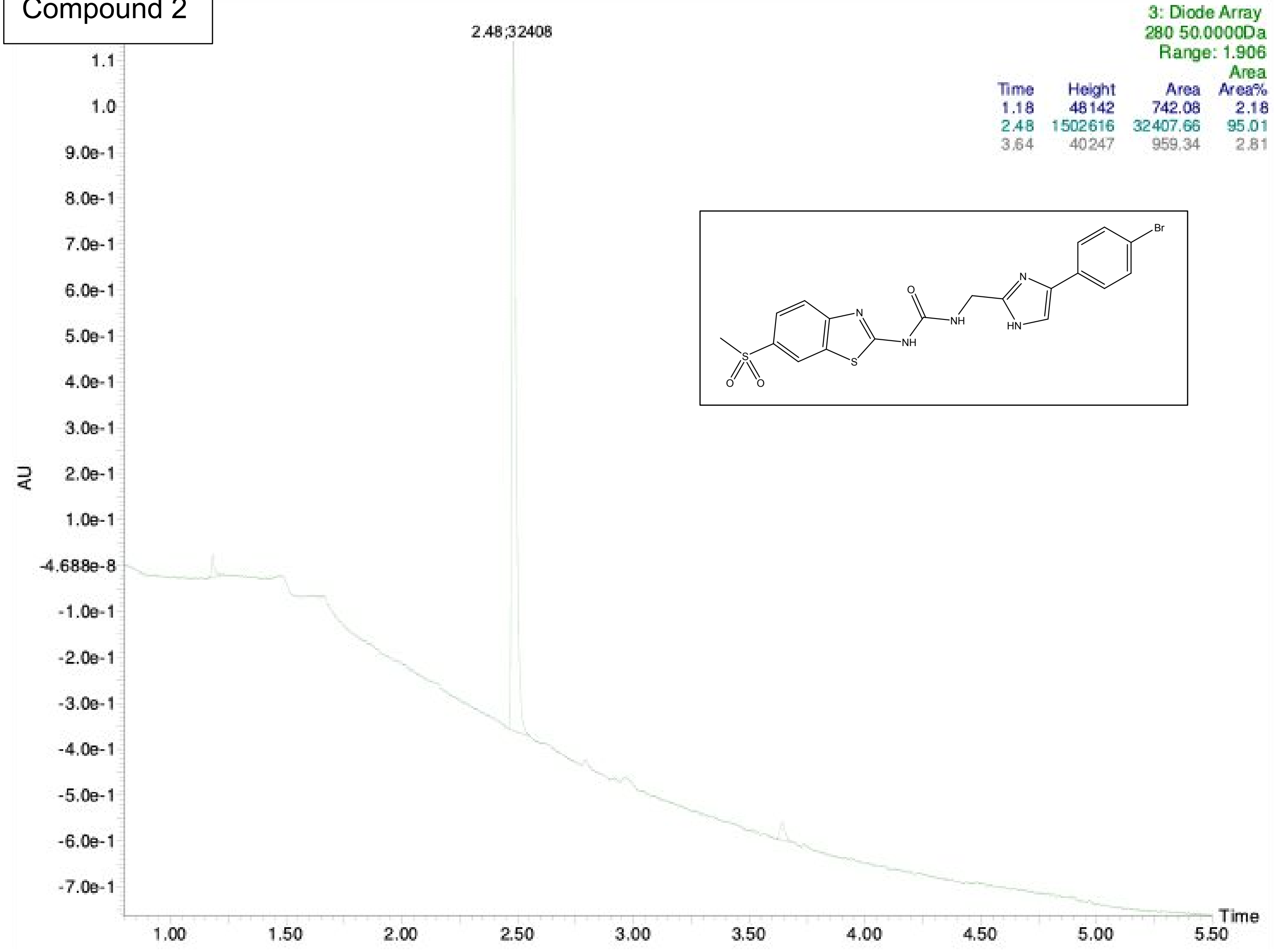
3: Diode Array
280 50.0000Da
Range: 1.906
Time Height Area Area%
1.18 48142 742.08 2.18
2.48 1502616 32407.66 95.01
3.64 40247 959.34 2.81
2.48;32408
AU
Time
1.00
1.50
2.00
2.50
3.00
3.50
4.00
4.50
5.00
5.50
1.1
1.0
9.0e-1
8.0e-1
7.0e-1
6.0e-1
5.0e-1
4.0e-1
3.0e-1
2.0e-1
1.0e-1
-4.688e-8
-1.0e-1
-2.0e-1
-3.0e-1
-4.0e-1
-5.0e-1
-6.0e-1
-7.0e-1
Br
N
O
NH
HN
S
O
O
S
NH

Compound 3

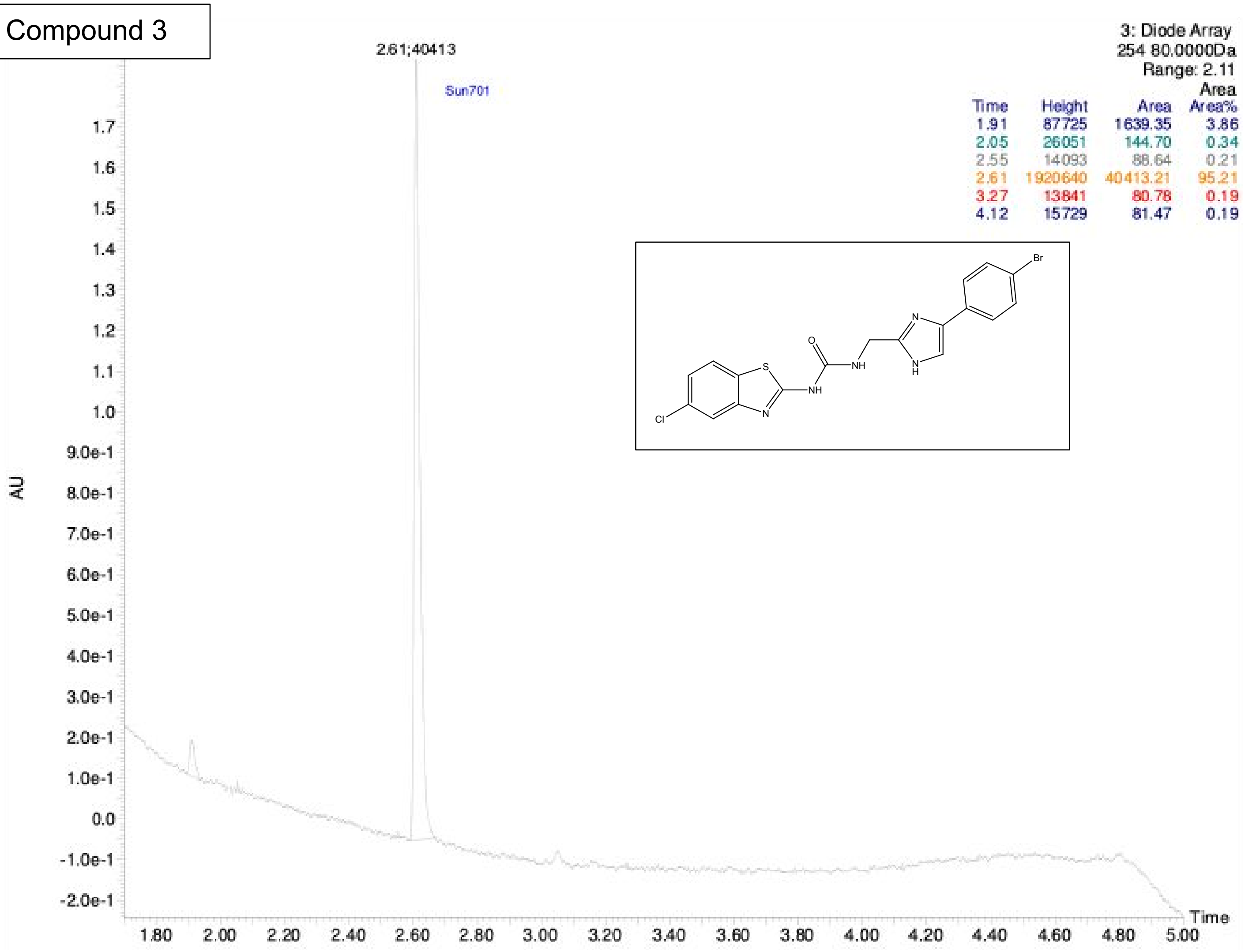

2.61;40413
Sun701
3: Diode Array
254 80.0000Da
Range: 2.11
Area
Time Height Area Area%
1.91 87725 1639.35 3.86
2.05 26051 144.70 0.34
2.55 14093 88.64 0.21
2.61 1920640 40413.21 95.21
3.27 13841 80.78 0.19
4.12 15729 81.47 0.19
Br
N
O
NH
S
N
H
NH
Cl
N
AU
1.7
1.6
1.5
1.4
1.3
1.2
1.1
1.0
9.0e-1
8.0e-1
7.0e-1
6.0e-1
5.0e-1
4.0e-1
3.0e-1
2.0e-1
1.0e-1
0.0
-1.0e-1
-2.0e-1
Time
1.80 2.00 2.20 2.40 2.60 2.80 3.00 3.20 3.40 3.60 3.80 4.00 4.20 4.40 4.60 4.80 5.00

Compound 4

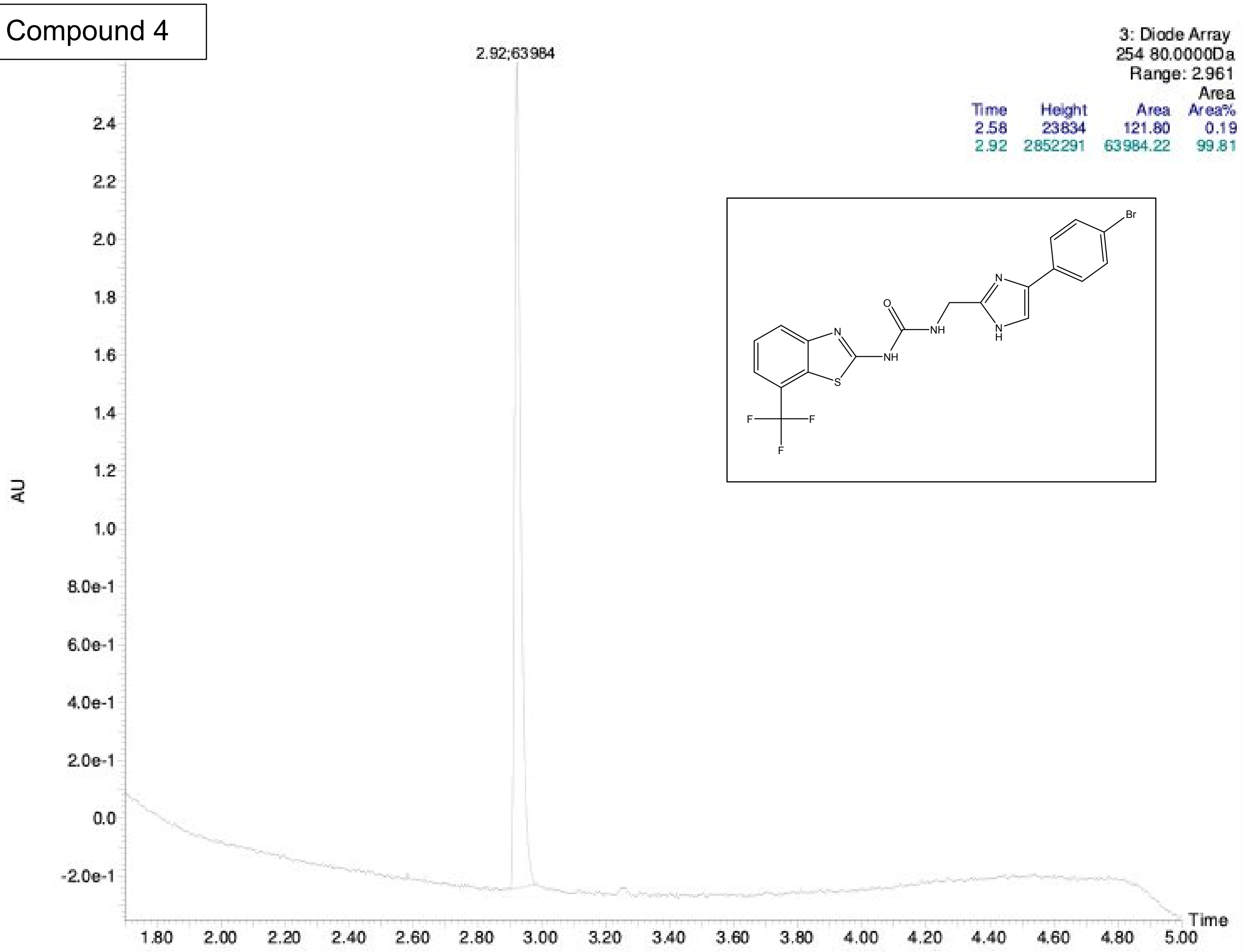

2.92;63984
3: Diode Array
254 80.0000Da
Range: 2.961
Time Height Area Area%
2.58 23834 121.80 0.19
2.92 2852291 63984.22 99.81
AU
Time
Br
N
H
NH
O
NH
N
S
F

Compound 5

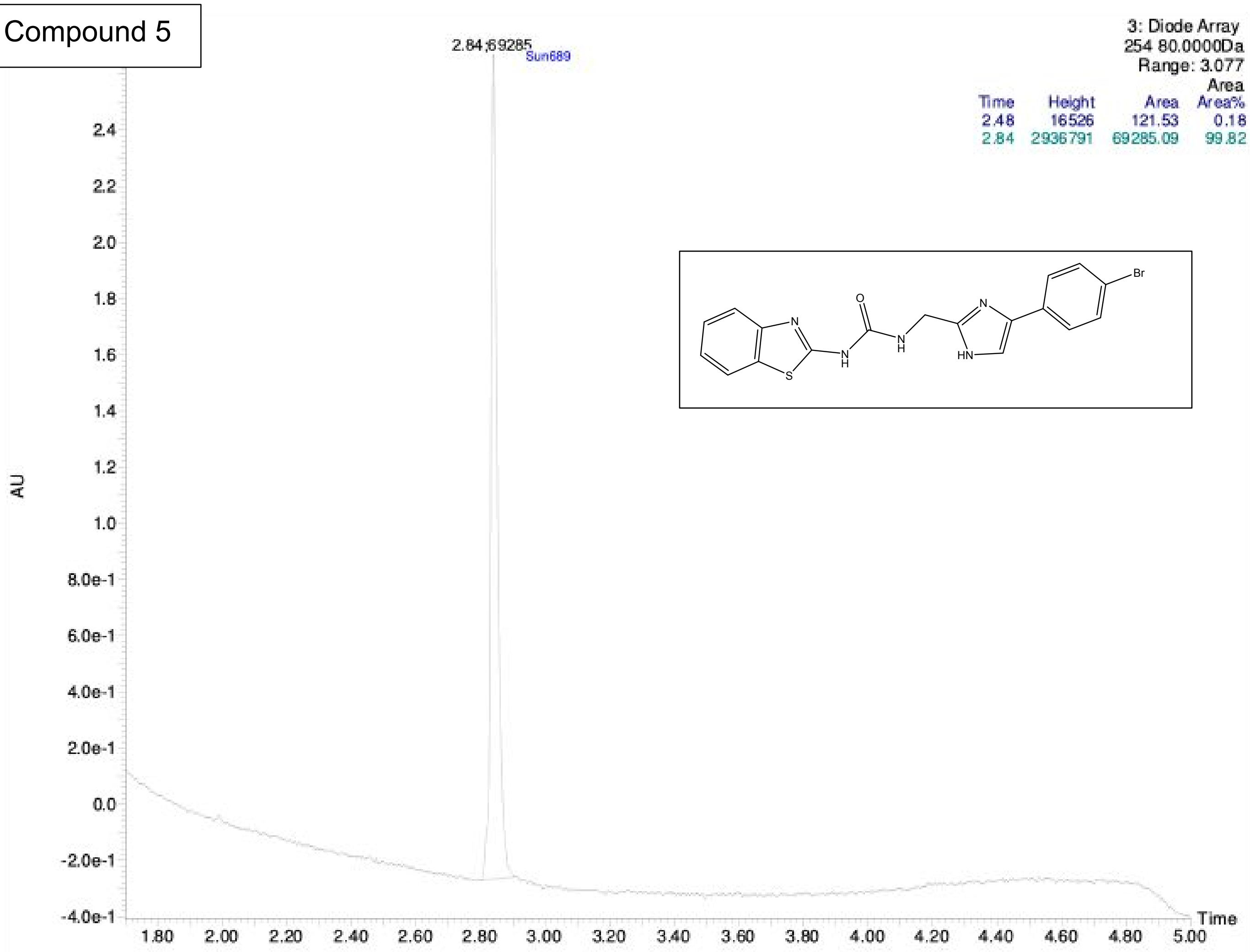

3: Diode Array
254 80.0000Da
Range: 3.077
Area
Time Height Area Area%
2.48 16526 121.53 0.18
2.84 2936791 69285.09 99.82
2.84;69285
Sun689
AU
Time
2.4
2.2
2.0
1.8
1.6
1.4
1.2
1.0
8.0e-1
6.0e-1
4.0e-1
2.0e-1
0.0
-2.0e-1
-4.0e-1
1.80
2.00
2.20
2.40
2.60
2.80
3.00
3.20
3.40
3.60
3.80
4.00
4.20
4.40
4.60
4.80
5.00
N
S
O
N
H
N
H
N
HN
Br

Supporting Tables

Table S1. Common commensal and pathogenic organisms Enoyl ACP Reductases.

**Body Site**	**Common Commensal Organisms**	**Enoly ACP Reductase Expressed**
**Skin**	*Staphylococcus epidermidis*	FabI[1]
**Skin**	*Corynebacterium* spp.	Type I fatty acid synthase (FAS-I)[2]
**Oral**	*Neisseria* spp.	FabI[3]
**Oral**	*Streptococcus mitis*	FabK[4]
**Gastrointestinal Tract**	*Bacteroides* spp.	FabI, FabK[5]
**Gastrointestinal Tract**	*Lactobacillus* spp.	FabI, FabL[6]
**Gastrointestinal Tract**	*Enterococcus faecalis*	FabI (predominately), FabK[7]
**Body Site**	**Common Pathogenic Organisms**	**Enoly ACP Reductase Expressed**
**Oral**	*Streptococcus mutans*	FabK[8]
**Oral**	*Porphyromonas gingivalis*	FabK[9]
**Oral**	*Treponema denticola*	TER[10]
**Oral**	*Fusobacterium nucleatum*	FabK[11]
**Oral**	*Prevotella intermedia*	FabI[12]
**Oral**	*Actinomyces israelii*	FabI[13]

Table S2. Data collection and refinement statistics.

	*Fn*FabK-*Compound 1*
**Wavelength**	0.9201
**Resolution range**	52.27 - 2.25 (2.31 - 2.25)
**Space group**	P 1 21 1
**Unit cell**	52.272 194.081 166.818 90 90.22 90
**Total reflections**	1119542 (80528)
**Unique reflections**	156928 (11137)
**Multiplicity**	7.1 (7.2)
**Completeness (%)**	99.59 (98.51)
**Mean I/sigma(I)**	3.73 (0.67)
**Wilson B-factor**	33.09
**R-merge**	0.3685 (2.09)
**R-meas**	0.3979 (2.252)
**R-pim**	0.1492 (0.8358)
**CC1/2**	0.984 (0.492)
**CC***	0.996 (0.812)
**Reflections used in refinement**	156290 (10971)
**Reflections used for R-free**	1985 (138)
**R-work**	0.2144 (0.3036)
**R-free**	0.2541 (0.3709)
**Number of non-hydrogen atoms**	30074
**macromolecules**	28211
**ligands**	975
**solvent**	888
**Protein residues**	3777
**RMS (bonds)**	0.003
**RMS (angles)**	0.6
**Ramachandran favored (%)**	96
**Ramachandran allowed (%)**	3.84
**Ramachandran outliers (%)**	0.16
**Rotamer outliers (%)**	0.63
**Clashscore**	8.29
**Average B-factor**	40.04
**macromolecules**	39.71
**ligands**	49.53
**solvent**	40.05

Table S3. Known membrane lipid composition of FabK-expressing species studied.[14,15,16,17]

Bacterium	Saturated Fatty Acids	Unsaturated Fatty Acids	Branched-Chain Fatty Acids
Fusobacterium nucleatum	Yes (C14:0, C16:0)	Yes (C16:1, C18:1)	*No clear evidence*
Clostridioides difficile	Yes (diacyl and plasmalogen forms)	Likely via plasmalogens	Yes (isocaproate, 5-methylcaproate)
Porphyromonas gingivalis	Yes (sphingolipid-like lipids)	*No clear evidence*	Yes (isobranched DHCs)
Streptococcus pneumoniae	Yes (C16:0, C18:0)	Yes (C16:1, C18:1; varies by phenotype)	Yes (iso/anteiso forms from BCAA metabolism)

References

1. Rana, P., Ghouse, S. M., Akunuri, R., Madhavi, Y. V., Chopra, S., & Nanduri, S. (2020). FabI (enoyl acyl carrier protein reductase) - A potential broad spectrum therapeutic target and its inhibitors. *European journal of medicinal chemistry*, *208*, 112757. https://doi.org/10.1016/j.ejmech.2020.112757
2. Sacco, Emmanuelle et al. "The missing piece of the type II fatty acid synthase system from Mycobacterium tuberculosis." Proceedings of the National Academy of Sciences of the United States of America vol. 104,37 (2007): 14628-33. doi:10.1073/pnas.0704132104
3. Yao, Jiangwei et al. "Activation of Exogenous Fatty Acids to Acyl-Acyl Carrier Protein Cannot Bypass FabI Inhibition in Neisseria." *The Journal of biological chemistry* vol. 291,1 (2016): 171-81. doi:10.1074/jbc.M115.699462
4. Denapaite, Dalia et al. "The genome of Streptococcus mitis B6--what is a commensal?." PloS one vol. 5,2 e9426. 25 Feb. 2010, doi:10.1371/journal.pone.0009426
5. Radka, Christopher D et al. "The genome of a Bacteroidetes inhabitant of the human gut encodes a structurally distinct enoyl-acyl carrier protein reductase (FabI)." The Journal of biological chemistry vol. 295,22 (2020): 7635-7652. doi:10.1074/jbc.RA120.013336
6. Yao, Jiangwei et al. "Enoyl-Acyl Carrier Protein Reductase I (FabI) Is Essential for the Intracellular Growth of Listeria monocytogenes." *Infection and immunity* vol. 84,12 3597-3607. 18 Nov. 2016, doi:10.1128/IAI.00647-16
7. Zhu, Lei et al. "The two functional enoyl-acyl carrier protein reductases of Enterococcus faecalis do not mediate triclosan resistance." mBio vol. 4,5 e00613-13. 1 Oct. 2013, doi:10.1128/mBio.00613-13
8. Kim, Tae-O et al. "Purification, crystallization and preliminary X-ray diffraction analysis of enoyl-acyl carrier protein reductase (FabK) from Streptococcus mutans strain UA159." Acta crystallographica. Section F, Structural biology and crystallization communications vol. 68,Pt 3 (2012): 292-4. doi:10.1107/S1744309112000115
9. Hevener, Kirk E et al. "Expression, purification and characterization of enoyl-ACP reductase II, FabK, from Porphyromonas gingivalis." Protein expression and purification vol. 85,1 (2012): 100-8. doi:10.1016/j.pep.2012.07.003
10. Tucci, Sara, and William Martin. "A novel prokaryotic trans-2-enoyl-CoA reductase from the spirochete Treponema denticola." FEBS letters vol. 581,8 (2007): 1561-6. doi:10.1016/j.febslet.2007.03.013
11. Rutherford, Jacob T et al. "Evaluation of Fusobacterium nucleatum Enoyl-ACP Reductase (FabK) as a Narrow-Spectrum Drug Target." ACS infectious diseases vol. 10,5 (2024): 1612-1623. doi:10.1021/acsinfecdis.3c00710

12. Karched, Maribasappa et al. “Proteomic analysis of the periodontal pathogen Prevotella intermedia secretomes in biofilm and planktonic lifestyles.” Scientific reports vol. 12,1 5636. 4 Apr. 2022, doi:10.1038/s41598-022-09085-0
13. Gago, Gabriela et al. “Fatty acid biosynthesis in actinomycetes.” FEMS microbiology reviews vol. 35,3 (2011): 475-97. doi:10.1111/j.1574-6976.2010.00259.x
14. Moye, Z. D., Valiuskyte, K., Dewhirst, F. E., Nichols, F. C., & Davey, M. E. (2016). Synthesis of sphingolipids impacts survival of *Porphyromonas gingivalis* and the presentation of surface polysaccharides. Frontiers in Microbiology, 7, 1919.
15. Aricha, B., Fishov, I., Cohen, Z., Sikron, N., Pesakhov, S., Khozin-Goldberg, I., Dagan, R., & Porat, N. (2004). Differences in membrane fluidity and fatty acid composition between phenotypic variants of *Streptococcus pneumoniae*. Journal of Bacteriology, 186(14), 4638–4644.
16. Zbylicki, B. R., Cochran, S., Weiss, D. S., & Ellermeier, C. D. (2025). Identification of two glycosyltransferases required for synthesis of membrane glycolipids in *Clostridioides difficile*. *mBio*, 16(3).
17. Jantzen, E. and Hofstad, T. (1981) Fatty acids of Fusobacterium Species: Taxonomic Implications. Microbiology, 123(1), 163-171.
